# Principal bundle geometry of qualia: Understanding the quality of consciousness from symmetry

**DOI:** 10.1093/pnasnexus/pgag261

**Published:** 2026-09-08

**Authors:** Masafumi Oizumi, Chanseok Lim, Ryota Kanai

**Affiliations:** Graduate School of Arts and Science, The University of Tokyo, Tokyo, Japan; Graduate School of Arts and Science, The University of Tokyo, Tokyo, Japan; Araya Inc., Tokyo, Japan

**Keywords:** equivariance, neural representational structure, principal bundle, qualia structure, symmetry

## Abstract

Qualia, the subjective qualities of experience, pose a fundamental challenge to scientific explanation. A promising approach is to characterize qualia by their relational structures rather than their intrinsic nature. Assuming the structure of qualia is characterized by the geometry of neural representations, we posit that this geometry is fundamentally organized by symmetry. We propose that the principal bundle, induced by a symmetry group *G* acting on a neural network’s state space, is the essential mathematical object uniquely characterizing this geometric structure. The core mathematical principle is that, for a *G*-equivariant network, its state space partitions into a quotient space *Q* and orbits induced by the group *G*. This framework reveals a three-level hierarchy for characterizing qualia. First, the choice of symmetry group *G* constrains the fundamental nature of the qualia modality (e.g. vision vs. audition). Second, we identify a qualia signature with a point in the quotient space *Q* (e.g. “cat”), representing the invariant identity of a percept. Third, we identify a qualia attribute with a position on a specific orbit (e.g. the cat’s location), representing its continuous variations. This framework uncovers a powerful duality: the geometry of the orbits is rigid, inheriting the structure of the group *G*, which explains the stable relational structure of qualia attributes. In contrast, quotient-space geometry is plastic, allowing the relationships between qualia signatures to be shaped by learning. This geometric hierarchy provides a unified language for explaining the structure of perceptual experience. Furthermore, our framework generates empirical predictions and a clear research strategy for characterizing qualia and comparing qualia across individuals.

Significance Statement Why does seeing a color feel fundamentally different from hearing a musical note? We cannot directly measure what an experience feels like, but we can measure how similar one experience feels to another. These similarity patterns define the relational structure of subjective experience. The structure differs across sensory modalities: color hues often exhibit circular structure, while musical pitches often exhibit helical structure. We propose that these patterns arise from symmetries that emerge in how the brain encodes sensory inputs, and that a single mathematical framework, principal bundle geometry, captures this organization. The framework yields testable predictions about which features of experience are universal across people and which differ between them.

## Introduction

The qualitative nature of our conscious experience, qualia, presents a profound scientific puzzle, organized across multiple levels of distinction between our experiences. In our usage, the term “qualia” is broadly defined as the properties of conscious states that characterize what it is like to have them, such as “what it is like to see red” and “what it is like to hear a C note” (1). At the most fundamental level, why do visual experiences feel distinctly visual and auditory experiences distinctly auditory? Within vision alone, why does perceiving the color “red” have its own unique character, distinct from “green”? How can we perceive a single, invariant object, such as a red apple, while its specific attributes, such as its location in our visual field or its orientation, continuously vary? Explaining how our brains generate such distinctive qualities of the experiences, qualia, is a central challenge of objective scientific investigation (2–7). Current theories of consciousness often focus on identifying the neural correlates or the functional roles of conscious states (8–10), proposing various functional mechanisms and systems-level properties of neural activity that are thought to be necessary and sufficient for conscious perception (11–17). However, many of these theories largely do not specify the principles that determine why a particular neural activity pattern should feel like “seeing red” as opposed to “hearing a C note,” nor do they explain the underlying relational structure that organizes these experiences.

**Figure 1:**
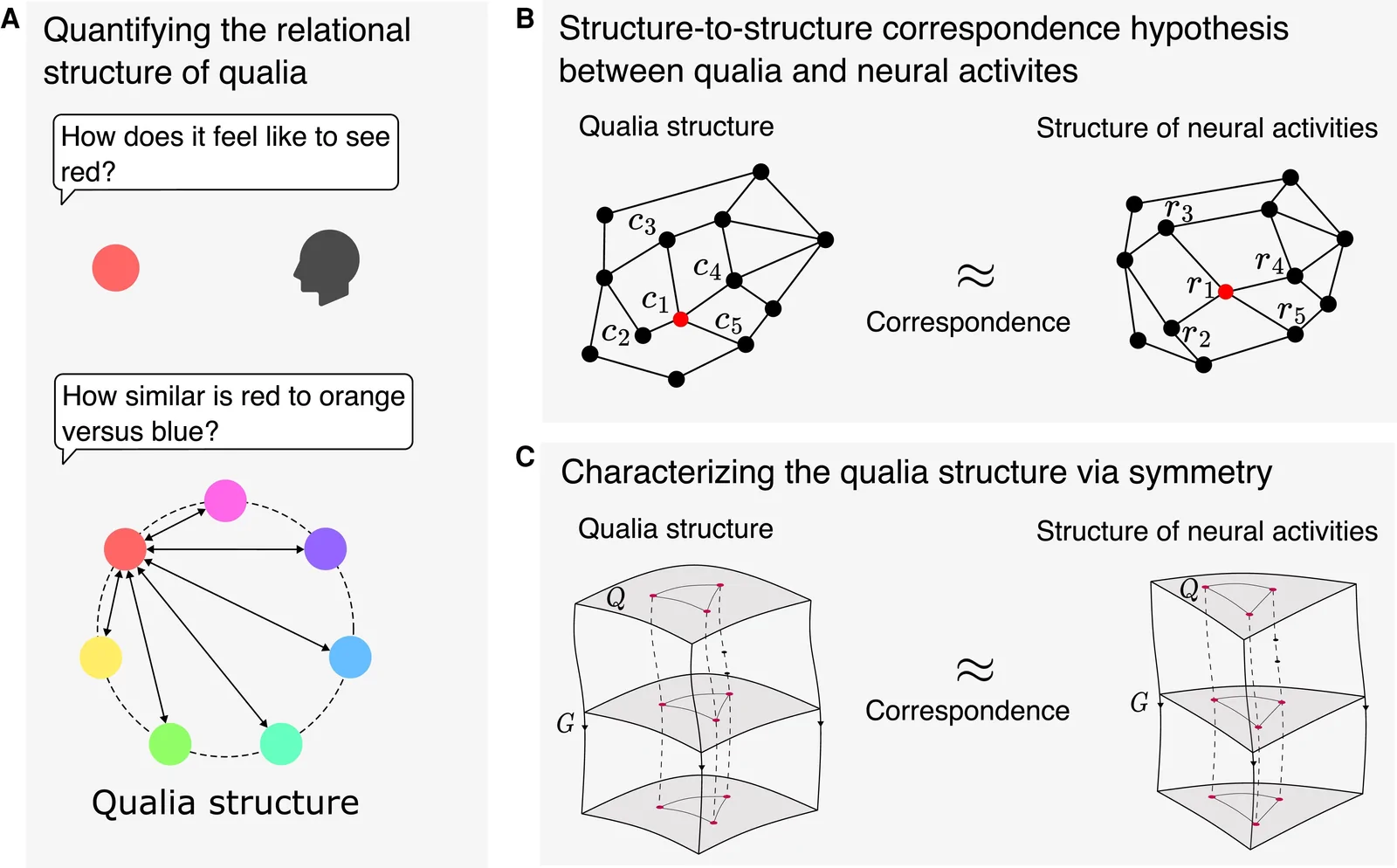
The three key components of the proposed theoretical framework for bridging qualia and neural activities. A) Quantifying the relational structure of qualia. For example, in the case of color experiences, rather than attempting to capture the intrinsic feeling of “seeing red” in isolation, we can empirically measure its perceived similarity to other experiences of colors. The relational structure among many different experiences is what we define as a “qualia structure.” Each point in a qualia structure represents a quale elicited by a particular stimulus, and the distance between points represents dissimilarity between the corresponding qualia. B) The hypothesis of structure-to-structure correspondence. Each point in qualia structures $c_{i}$ represents a particular quale and the distance between points represent dissimilarity between qualia. The point $r_{i}$ in the structure of neural activities represents the corresponding neural activity that occurs when the person has this particular experience $c_{i}$. and the distance between the points represent dissimilarity between the neural activities. Our central working hypothesis is that there is a faithful correspondence between the qualia structure and the relational structure of the underlying neural activities. This hypothesis provides the crucial link that allows the structure of neural representations to serve as a model for the structure of subjective experience. C) Characterizing the qualia structure via symmetry. Accepting the structure-to-structure correspondence hypothesis allows us to characterize the qualia structure by analyzing the geometry of neural representations. Our approach argues that this geometry is shaped by symmetry. The principal bundle structure, which naturally emerges when a network learns to process its inputs equivariantly, provides a concrete mathematical model for this neural organization. By extension, it offers a predictive model for the structure of qualia.

To bridge the explanatory gap between neural activity and qualia, our framework builds a logical bridge from subjective experience to neural geometry in three steps (Fig. 1). First, we adopt a structural approach that seeks a characterization of qualia, grounded in the geometric structure of a system (18–24). Rather than directly asking about the ineffable intrinsic quality of an experience, this approach quantifies the relational structure of qualia—for instance, measures the perceived similarity of “red” to “orange” vs. “blue” (24) (Fig. 1A). Second, to connect this empirically accessible “qualia structure” to the neural activity, we assume a working hypothesis of structure-to-structure correspondence (Fig. 1B): that the geometry of subjective experience is faithfully mirrored by the geometry of its underlying neural representation. Third, accepting this correspondence defines our core scientific strategy: to characterize the geometry of neural representations (Fig. 1C). This focus on the geometry of computational architecture is strongly motivated by pioneering “rewired ferret” experiments, which suggest that the character of a qualia modality, such as vision or audition, is determined not by a brain area’s location, but by the computational structure of its neural circuits (25–28).

**Figure 2:**
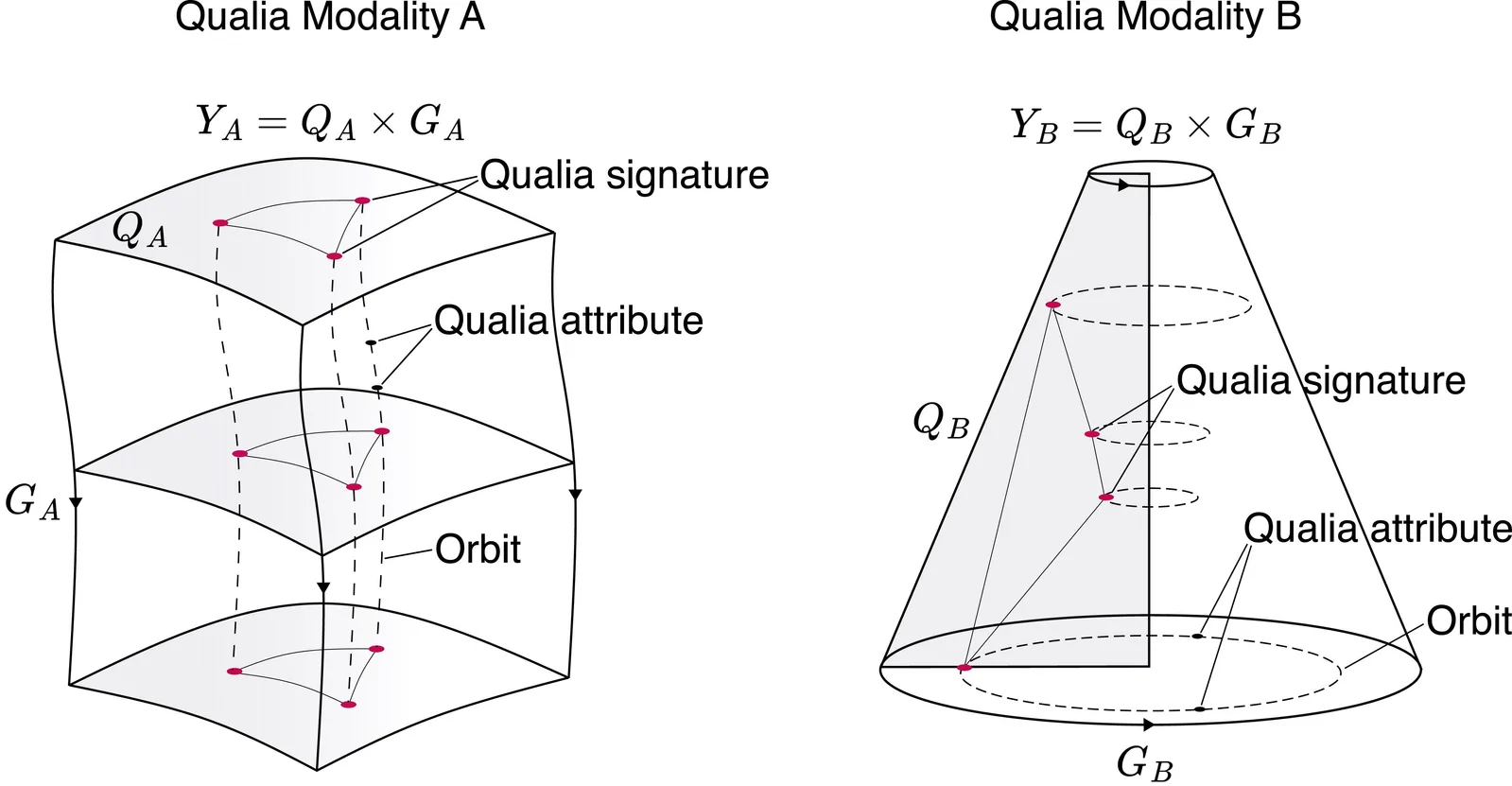
The principal-bundle geometry of qualia. Qualia modalities A and B differ in their groups $G_{A}$ and $G_{B}$, which reflect different invariant signal transformations. The bundle for each qualia modality is the product of the quotient space *Q* and the symmetry group *G* (arrows orthogonal to *Q*), which encodes transformations that leave the identity in *Q* invariant. The multiple shaded planes shown are cross-sections, each representing a copy of the quotient space. Thus, the bundle structure of each modality varies according to the transformations that preserve the identity in *Q*. Qualia signatures are defined by red points in *Q*. These points represent the *G*-invariant identities of different percepts (e.g. the signatures “cat” and “dog”). Qualia attributes are defined by points on a specific orbit (a dashed line). These represent the continuous, *G*-variant parameters of a percept (e.g. the specific locations of the “cat”).

Building on this strategy, we propose that the fundamental principle organizing neural geometry is symmetry, a concept that has proven foundational to learning in both biological and artificial systems (29–32). The application of group theory to characterize perceptual invariants has a long history (33–37). Our goal is to develop a general theory applicable across diverse sensory modalities by leveraging modern computational neuroscience and geometric deep learning. To formalize this theory, we employ the mathematical framework of a principal bundle (38, 39) (Fig. 2). This structure naturally arises from any equivariant neural encoder (*G*), which partitions the neural state space (*Y*) into two components: orbits (O), representing continuous perceptual variations generated by the group’s action, and the quotient space (Q=Y/G), representing dimensions of variation orthogonal to the group action. Decomposing a representation space into the orbits of a group action and a quotient space of invariants is a central concept in modern geometric theories of perception and recognition (40–46). Throughout, we treat exact *G*-equivariance as an idealized structural limit, not a literal claim about biology: real perceptual systems exhibit only approximate, task- and scale-dependent constancy, and the literal program of imposing exact group structure on perception has accordingly been critiqued as too idealized if read as a description of cortical processing (47). The empirical question we foreground is therefore which approximate symmetries neural systems realize and how strongly, with the ideal bundle geometry serving as a measurable baseline against which biological deviations can be quantified (discussed in detail in the Approximate and emergent equivariance section below).

Our central proposal is that this principal bundle geometry uniquely characterizes the relational structure of qualia. Given this geometry, the three-level hierarchy of qualia naturally emerges (Fig. 2).

- **Qualia modality**: Constrained by the algebraic structure of the symmetry group *G* itself. The nonisomorphic nature of the groups that govern different senses (e.g. SO(2) for hue vs. R for pitch) accounts for the fundamental differences between sensory worlds.
- **Qualia signature**: Defined by a point in the bundle’s base space, the quotient space (Q=Y/G). This represents the *G*-invariant identity of a percept (e.g. the signature “cat”).
- **Qualia attribute**: Defined by a point on a specific orbit or fiber O⊂Y. This represents the continuous, *G*-variant parameters of a percept (e.g. the specific location of the “cat”).

The scope of our framework is to provide a structural characterization of perceptual qualia in systems that independently support conscious perceptual experience, rather than to propose equivariance as a criterion for consciousness. We do not claim that any artificial or biological system whose internal representations satisfy the relevant symmetries is thereby conscious (sufficiency), and we do not claim that consciousness requires symmetry-structured neural representations (necessity). Whether a given system supports conscious experience is treated as a question prior to, and outside the scope of, the present framework; within that scope, principal-bundle geometry is offered as a structural description of how perceptual qualia are organized in candidate conscious systems, not as a criterion that confers consciousness on systems exhibiting it. We also note that under the broad definition of qualia (1), we do not attempt to resolve disputes about whether qualia are intrinsic properties or relational properties (48).

The primary contribution of this framework is the exposure of a fundamental duality: the geometry of qualia attributes is rigid, while the geometry of qualia signatures is plastic. The orbits (O), which encode attributes, inherit their topological structure directly from the fixed, universal symmetry group (*G*). In stark contrast, the quotient space (*Q*), which encodes signatures, is a learnable manifold whose geometry is actively shaped by experience to reflect the statistical structure of the environment. This “rigid attribute, plastic signature” principle provides a powerful lens for understanding differences in qualia structures across individuals. For instance, if two people acquire the same equivariance properties, the relational structure of their qualia attributes must be topologically equivalent. In contrast, there is no theoretical guarantee that the geometric relationships between their qualia signatures are similar, as this structure is shaped by each individual’s unique learning history.

The following sections will introduce the underlying mathematical concepts of our theory and derive the consequences of principal bundle geometry that lead to this fundamental duality. We will then outline a research strategy using principal bundle geometry and its duality to fully characterize qualia structures and compare them across individuals.

## Symmetry groups and equivariance of neural representation

As illustrated in Fig. 1, the core strategy of our framework is to characterize the structure of neural activity via “symmetry,” providing a bridge to the structure of qualia. To formalize this characteristic structure, we will now introduce the essential mathematical concepts at its foundation. This section introduces symmetry groups, group actions, and the principle of equivariance. The following section will then demonstrate how these concepts give rise to the specific geometric structures of orbits and quotient spaces, which are central to our theory.

Before proceeding, we clarify what we mean by “symmetry.” Symmetry in our framework does not refer to geometric or visual symmetry of the perceptual space as embedded in some empirical metric: perceptual spaces are well known to exhibit pronounced asymmetries and need not look “round” or “balanced” in any visual sense. Symmetry refers instead to the action of a group *G* under which the neural encoder is (approximately) equivariant. Such a *G*-action need not be global over an entire perceptual space; it may be local, regime-restricted, or apply only to a subset of dimensions, and observed asymmetries between dimensions on which *G* acts and those on which it does not are exactly the orbit/quotient distinction the framework predicts. Importantly, the relevant *G*-action is realized at the level of the neural encoder rather than as a symmetry of the physical stimulus space; sensory transduction and neural processing can induce effective symmetries (such as the circular structure of hue, which has no counterpart in physical wavelength space) and shared sensory physiology, not direct physical invariants, is what makes attributes universal across observers with comparable physiology.

### Symmetry groups

At the heart of our theory lies the concept of a symmetry group. Intuitively, symmetries describe transformations that leave a key aspect of an object or system unchanged. A group is the mathematical formalization of this concept. It is a set of transformations, denoted *G*, that includes an identity transformation (which does nothing), where any two transformations can be combined to form a third transformation, and every transformation has an inverse. For instance, the set of all possible translations of an image on a 2D plane forms a group because any two translations can be combined, and every translation has an inverse. This group can be denoted as $G=R^{2}$. Throughout this article, the symmetry group *G* is taken to be a Lie group, that is, a group that is simultaneously a smooth manifold with smooth group multiplication, acting smoothly on a smooth manifold *Y*. This differentiable structure underlies the formal propositions developed in the Supplementary material.

### Group actions

A group becomes meaningful when it acts on a space, meaning each transformation in the group maps points in the space to other points within the same space. This is how abstract symmetries manifest as concrete changes. In our framework, we consider two such actions:

1. **Action on the stimulus space (*X*):** This is the world of sensory inputs. For a stimulus x∈X (e.g. an image of a cat) and a group element g∈G (e.g. a specific translation), the action g⋅x produces the transformed stimulus (the translated image).
2. **Action on the neural state space (*Y*):** This is the world of neural activity. For a neural activity pattern y∈Y, a group element *g* also induces a transformation, denoted by ρ(g). The transformed neural state is thus ρ(g)y. The function *ρ* is called a representation of the group. It translates the abstract group elements into concrete operations within the neural state space.

### Equivariance: linking stimulus and neural symmetries

**Figure 3:**
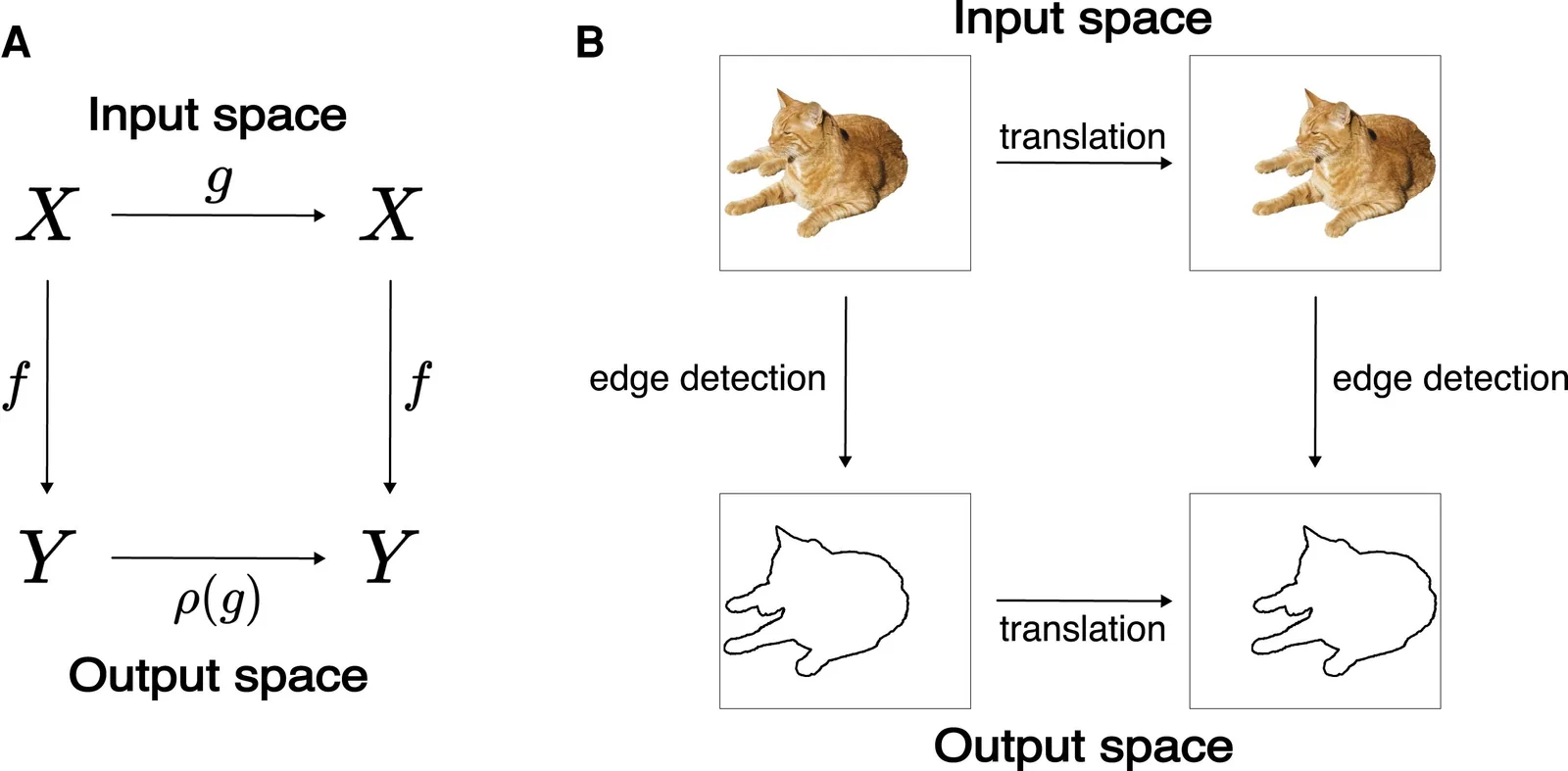
Definition and toy example of equivariance. A) Commutative diagram for (G,ρ)-equivariance. The input transformation of a group element *g* and the corresponding output transformation ρ(g) run horizontally, while the function f:X→Y (neural networks) acts vertically. The diagram commutes if and only if f(g⋅x)=ρ(g)f(x) for all g∈G,x∈X. B) A toy example illustrating equivariance in image processing. Applying a translation to the right on the input image, i.e. a lying cat, followed by edge detection, yields the same result as applying edge detection first, followed by a translation to the right on the output. This demonstrates that the edge detector *f* is equivariant with respect to the translation group.

The crucial link between the stimulus and the neural world is a property called “equivariance.” A neural encoder, f:X→Y, is said to be *G*-equivariant if the two paths, visualized in Fig. 3, lead to the same result:

- **Transform then encode**: Applying a transformation g∈G to the input *x* and then encoding the result corresponds to computing f(g⋅x).
- **Encode then transform**: Encoding the input *x* first and then applying the corresponding transformation to the neural representation corresponds to computing ρ(g)⋅f(x).

This principle of consistency is captured by the following equation:


(1) f(g⋅x)=ρ(g)⋅f(x)for all g∈G,x∈X

Intuitively, the property of equivariance forces the neural representation space *Y* to be organized by the symmetries of the group *G*. As will be elaborated later, this imposed geometric organization gives rise to a characteristic structure known as a principal bundle, which provides the mathematical foundation for our theory of qualia.

Note that invariance is a special case of equivariance in which the representation is the identity, i.e. f(g⋅x)=f(x). Characteristic principal bundle geometry does not appear in the case of invariance. Thus, this manuscript is mostly concerned with cases of equivariance where the group representation *ρ* is not the identity (more precisely, *ρ* acts freely on *Y*).

## Equivariance in neuroscience and AI

Neuroscientific evidence indicates that equivariance and invariance are canonical principles of information processing in the brain. The principle of progressing from equivariant to invariant representations, in particular, is a cornerstone of sensory processing. In the visual system, early areas like V1 contain retinotopic maps that represent stimuli in an explicitly equivariant manner, where a translation of a stimulus on the retina causes a corresponding translation of the activity pattern on the cortex (49–51). As information progresses to higher-order areas like the inferior temporal (IT) cortex, neurons develop representations of object identity that are remarkably invariant to changes in position, scale, and viewpoint (52–57). This progression is a general feature across modalities, with the auditory system showing equivariance to pitch transposition in tonotopic maps (58) and the somatosensory system showing equivariance to the location of tactile stimuli (59). Crucially, this development of invariance does not mean that attribute information is discarded; neural populations continue to encode properties like position and color in a manner that varies systematically with transformations, preserving the information for a complete perceptual experience (60–63).

Inspired by these biological principles, AI has achieved tremendous success by incorporating symmetry into network architectures. The most prominent example is the convolutional neural network (CNN), whose shared-weight structure explicitly enforces translational equivariance, a powerful inductive bias (64, 65). This idea has been generalized in Geometric Deep Learning, which develops models that respect other symmetries like rotation by incorporating group theory (32, 42, 66–68). This convergence has created a powerful synergy where equivariant deep learning models are now state-of-the-art tools for understanding cortical and subcortical processing in vision and audition (69–75). The ability of these AI models to explain neural activity in the ventral stream is strongly correlated with how well they learn invariant object representations (76, 77), underscoring that equivariance is a canonical principle of both biological and AI.

The emergence of equivariance in neural systems can be understood through the principles of efficient coding and statistical learning (29–31, 40). Natural environments exhibit a rich symmetrical structure. For example, objects appear at different locations, orientations, and scales, yet maintain their identity. Neural systems that learn to extract these invariant features while preserving information about the transformations achieve a more efficient representation, requiring fewer parameters to encode the world. This pressure for efficiency, combined with the statistical regularities of sensory input, naturally drives neural networks toward equivariant solutions. In biological systems, additional constraints such as metabolic costs and wiring economy further favor such symmetry-respecting architectures. Thus, equivariance emerges not as an imposed design choice, but as an optimal solution to the fundamental challenge of learning structured representations from symmetrical environmental data.

## The predicted geometry of qualia: the principal bundle

When a neural encoder is equivariant with respect to a symmetry group *G*, this symmetry naturally organizes or “decomposes” the neural state space *Y* into a structure known as a “principal bundle” (see Supplementary material for a formal definition). Intuitively, symmetry itself is what creates structure; by imposing constraints on the geometry of the neural representation, it forces an otherwise uniform space to acquire a specific, inevitable organization. This structure consists of two fundamental components: “orbits” and a “quotient space” (Fig. 2).

The central prediction of our framework is that this principal bundle geometry provides the mathematical characterization of qualia, assuming the structure-to-structure correspondence hypothesis (Fig. 1C). Specifically, we propose that it organizes subjective experience into the following three-level hierarchy:

### Symmetry groups: the identity of qualia modalities

At the most fundamental level, we propose that the algebraic structure of the symmetry group *G* itself constrains the structural organization of a qualia modality. Assume that neural encoders for different sensory modalities, such as vision and audition, acquire equivariance with respect to different symmetry groups, $G_{A}$ and $G_{B}$. For generality, each of these can be a composition of multiple symmetry groups; the core assumption is that the overall structure of $G_{A}$ is nonisomorphic to $G_{B}$. Because orbits inherit their topology directly from their generating group as formalized later, these different groups will induce orbits with fundamentally different topological structures (Fig. 2). Conversely, all orbits generated by the same group are topologically equivalent to each other. We posit that this difference in orbit topology, rooted in the nonisomorphic nature of the underlying symmetry groups, corresponds to the fundamental phenomenological difference between sensory worlds.

### Orbits: the geometry of qualia attributes

For any given neural state *y*, the set of all states reachable by applying transformations from *G* forms the orbit of *y*. Mathematically, this is written as


(2) O(y)={ρ(g)⋅y∣g∈G}.

We propose that an orbit O(y) is a submanifold within the neural space that can be thought of as a pathway containing all possible changes in the attributes of an experience corresponding to *y*, called “qualia attributes” (Fig. 2). Thus, a specific point on an orbit represents a particular qualia attribute, while the entire orbit represents the full range of possible attributes for a given signature. For instance, one orbit could consist of all the neural states corresponding to a specific cat, seen at every possible position in the visual field. Moving along an orbit changes the sensory parameters of the experience, i.e. its attributes, such as location or orientation, but not the core identity of what is being perceived.

### Quotient space: the geometry of qualia signatures

If we treat each entire orbit as a single abstract point, the collection of all these points forms a new space called the quotient space, written as Q=Y/G (Fig. 2). This is the space that remains after we “quotient out,” or disregard, the variations that occur within each orbit. There is a natural projection map, π:Y→Q, that takes any point *y* in the full neural space and identifies which orbit it belongs to.

We propose that the quotient space *Q* is the space of “qualia signatures” (Fig. 2). Each point in *Q* represents a distinct, G-invariant identity of an experience, such as “cat” and “dog,” without regard for their specific, variable attributes.

## Phenomenology of qualia signatures and attributes in different modalities

The principal-bundle framework provides a unified explanation for the structure of diverse sensory experiences. Here, we illustrate how the geometric decomposition maps onto the phenomenology of different qualia modalities, such as vision and audition. These examples demonstrate that the specific algebraic structure of the relevant symmetry group *G* constrains the modality, while the corresponding orbits and quotient spaces define its attributes and signatures. This theoretical approach finds strong support in a variety of findings from perceptual neuroscience, which reveal how neural systems build representations that are tolerant to some transformations while remaining sensitive to others (31, 78).

The examples below should not be read as claims that equivariance has been rigorously established for these modalities. The relevant neural correlates have not yet been definitively identified, and the corresponding perceptual symmetries have not been formally derived from psychophysical data. We treat these examples as likely and plausible candidates, motivated by accumulated evidence from neuroscience and psychophysics, that the framework is intended to organize and refine through the bidirectional, iterative methodology described later in the section “Approximate and emergent equivariance.”

### Visual objects under translation

**Figure 4:**
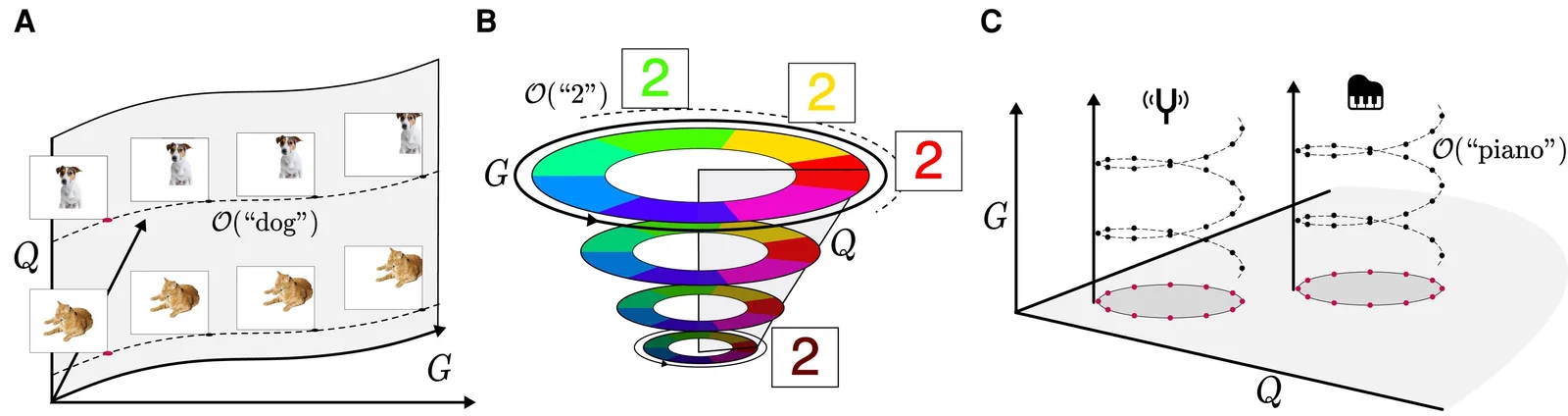
Mapping principal bundle structures onto the phenomenological structures of experiences in different modalities. A) The translation group in visual objects. Qualia signatures in *Q* correspond to object identities (“dog” and “cat”). Qualia attributes correspond to changes in a curved line orbit (a dashed line, e.g. O (“dog”)) under the action of *G*, representing spatial instantiations of the same signature (“the dog at the center”). B) The hue-rotation group G=SO(2) in colors. Qualia signatures in *Q* correspond to changes in color saturation and lightness. Qualia attributes correspond to changes in a circular orbit (rainbow ring along with a dashed line, e.g. O (“2”)), representing hue variations of the same object at fixed saturation and lightness. C) The pitch shift group G=R in sounds. Qualia signatures in *Q* correspond to timbre identities (“pure tone” on the left and “piano note” on the right). These signatures form pitch-class circles for specific timbres (red markers). Qualia attributes correspond to changes in a helical orbit, e.g. O(“piano”), representing different pitches of the same timbre.

This framework explains how we perceive an object’s identity as distinct from its spatial location (Fig. 4A). Under the translation group $G=R^{2}$, the object’s identity (e.g. “dog”) is the qualia signature, a point in the quotient space invariant to position. Its specific location is the qualia attribute, a point on the corresponding orbit. As an object moves, its neural state glides along this orbit, changing its perceived attribute while the signature remains constant. This decomposition parallels the “two-streams hypothesis,” where the ventral stream processes object identity and the dorsal stream processes location (79, 80), and is supported by findings of position-tolerant representations in the inferior temporal cortex (52, 55) and the role of location as a perceptual cue (81).

### Colors under hue rotation

The circular nature of color perception (Fig. 4B) is modeled by the rotation group G=SO(2). In this model, hue is the qualia attribute, corresponding to a position on a circular orbit ($S^{1}$), while saturation and lightness are the qualia signatures, represented in the quotient space. As a color’s hue changes, its neural state moves along the circular orbit (e.g. through the rainbow), but if its saturation decreases, the state moves toward the central axis, changing the signature from “chromatic” to “achromatic.” This geometry is supported by perceptual maps of color similarity (82) and is illustrated by the circular arrangement of hue-tuned neurons in visual area V4 (83, 84).

### Sounds under pitch shift

The perception of a stable sound identity (timbre) across variations in pitch (Fig. 4C) is modeled by the pitch shift group G=R. Timbre (e.g. “piano note”) acts as the qualia signature, a point in the quotient space that is tolerant to pitch transposition, while the specific pitch is the qualia attribute, a position along a line or helical orbit. Thus, changing the pitch of a piano note corresponds to moving along an orbit, whereas changing the sound from a pure tone to a complex one constitutes a jump to a different orbit. This model is consistent with our ability to recognize instruments regardless of the note played (85) and with evidence that the brain can disentangle pitch and timbre, despite their perceptual interaction (86–89).

## The duality of qualia geometry: rigid attributes and plastic signatures

**Figure 5:**
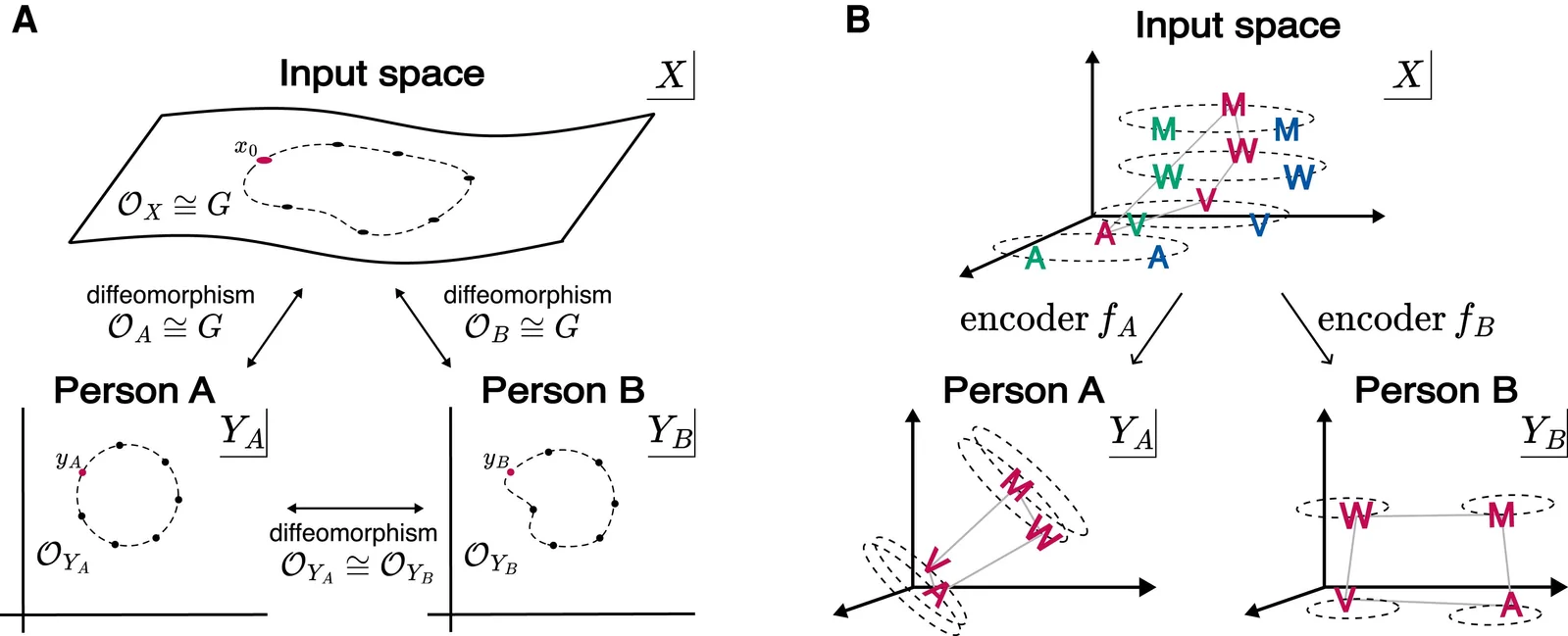
Rigidity of qualia attributes and plasticity of qualia signatures. A) Rigidity of qualia attributes. Any orbit formed by the group action *G* in the neural representation space *Y* is diffeomorphic to *G* (Supplementary material, Proposition S1). Orbits in person A and person B, $O_{Y_{A}}(y_{A})$ and $O_{Y_{B}}(y_{B})$, are diffeomorphic (Supplementary material, Proposition S2). This implies that the structure of qualia attributes, as represented by the structure of orbits, is topologically rigid and universal across subjects. B) Plasticity of qualia signatures. The symbols “A,” “M,” “V,” and “W” form orbits under the group action *G* in the input space *X*, which corresponds to color changes. A copy of the quotient space represents the relational structures of the red color symbols “A,” “M,” “V,” and “W,” for example. The relational structures formed by the red color symbols can differ between the input space and the neural representation space (Supplementary material, Proposition S5). Thus, in general, persons A and B have different relational structures in the quotient space (Supplementary material, Proposition S6). This implies that the structure of qualia signatures, as represented by the relational structure in the quotient space, is plastic and subject-specific.

Our geometric framework reveals a fundamental duality in the structure of subjective experience, which we formalize with a series of mathematical propositions: the relational structure of qualia attributes is rigid and universal, whereas the structure of qualia signatures is plastic and person-specific (Fig. 5). This duality provides a principled explanation for which aspects of consciousness are shared and which are unique to the individual. The formal mathematical proofs for these claims are detailed in the Supplementary material.

The rigidity of qualia attributes stems from their inheritance of structure from the effective symmetries the neural encoder realizes. As illustrated in Fig. 5A, when a transformation from a symmetry group *G* (such as translation) is applied in the input space, it creates a corresponding orbit of activity in the neural representation space *Y*. Our propositions first establish that this resulting orbit in the brain must have the same fundamental shape as the symmetry group itself. A key consequence, proven in a subsequent proposition, is that this structure must be universal across individuals. Even if person A and person B have very different neural implementations for processing visual information, the orbits representing the same attribute in their respective brains ($O_{Y_{A}}$ and $O_{Y_{B}}$) will be structurally equivalent—they are, in mathematical terms, diffeomorphic. This means that the subjective experience of continuous attributes, like the circular nature of hue or the planar structure of visual space, is not arbitrary but is a universal feature of perception shared across individuals. Further propositions show that under stricter neural conditions, this similarity becomes even stronger, preserving not just the overall shape but also the metric relationships (i.e. distances and angles) within the attribute space, up to a uniform distortion.

In stark contrast, the geometric relationships between different qualia signatures, for example, the perceived similarity between a “cat” and a “dog,” are not fixed by symmetry alone. Our propositions on plasticity demonstrate that this part of the neural geometry is learnable and can be arbitrarily shaped by experience. Figure 5B illustrates this concept. Consider a set of distinct inputs, here, the letters “A,” “M,” “V,” and “W,” that are invariant to a transformation like color change. These invariant identities are the qualia signatures, and their relationships define the geometry of the quotient space. The figure depicts how person A might learn a neural representation where “M” and “W” are perceived as similar (and thus are close together in their geometric space), while person B learns a different structure where “V” and “W” are perceived as similar. This difference arises because their unique experiences have shaped their conceptual maps differently. This leads to the critical insight that the conceptual maps built upon the universal attribute axes can differ dramatically from person to person. However, our framework also shows that for any given individual, this learned space is internally coherent; the relationship between “cat” and “dog” remains stable regardless of the viewpoint or context. Our framework thus formalizes the duality of a shared perceptual world governed by rigid physical laws and a private, plastic understanding of it.

## Comparing qualia structures across individuals

Beyond its theoretical value, our geometric framework provides a concrete empirical program to address a central challenge in consciousness science: determining whether two individuals have similar subjective experiences (24). While private experiences cannot be compared directly, the structural approach makes this problem tractable by shifting the focus from the experiences themselves to their relational structures. This reframes the challenge as a quantitative scientific question: how similar are the qualia structures of two different individuals?

**Figure 6:**
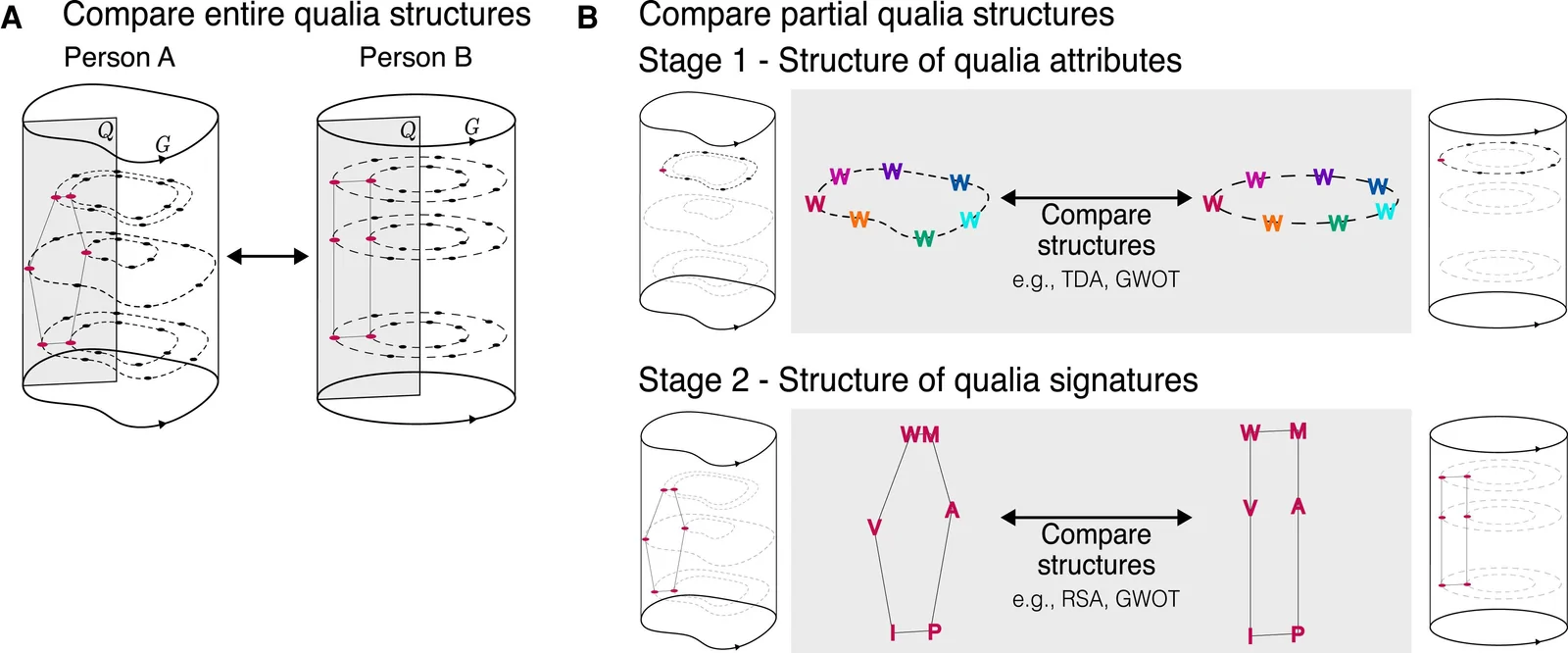
Two-stage protocol for comparing qualia structures across individuals.n A) Comparing entire qualia structures across individuals. The two cylinders represent the entire qualia structures of person A and person B. These structures are characterized by principal bundle geometry consisting of the product of the quotient space *Q* and the symmetry group *G*. B) Comparing partial qualia structures across individuals. A person’s entire qualia structure can be decomposed into two different types of structures: (1) the structure of qualia attributes, which are rigid and universal across individuals (e.g.“W,” “M,” “V,” “A,” “I,” “P” (representing object identities) under the group action *G* (color changes). The shaded planes are cross-sections showing copies of the quotient space *Q*. Red and black dots denote example qualia signatures, with the red dots highlighting particular signatures on the cross-sections. These qualia structures can be compared using several representative tools. For stage 1 (the structure of qualia attributes), tools that probe topological similarity are appropriate, such as TDA) and GWOT. For stage 2 (the structure of qualia signatures), tools that probe geometric similarity are appropriate, such as RSA and GWOT.

Our framework, based on principal bundle geometry, offers novel approaches to addressing this question. First, we propose that it is essential to compare the qualia structures as a whole by considering both qualia attributes along the orbits and qualia signatures in the quotient space (Fig. 6A). To conclude that qualia structures are equivalent across individuals, we must demonstrate that the whole qualia structures align very well.

In addition to this holistic comparison, the core prediction of our framework, i.e. the duality of rigid, universal attributes and plastic, person-specific signatures, motivates a more nuanced, two-stage protocol (Fig. 6B). This approach analyzes the two structural components separately, allowing for a deeper comparison. For example, if two individuals’ qualia structures do not align as a whole, this protocol can help determine whether the discrepancy stems from their representation of attributes, signatures, or both.

### Stage 1: the universal structure of qualia attributes

This stage tests the strong theoretical prediction that the structure of qualia attributes, represented by orbits, is universal across individuals. Because shared sensory physiology produces shared effective symmetries across observers, their neural representations of these symmetries should share a common geometric shape (Fig. 6B). To verify this, we can use tools from Topological Data Analysis (TDA), such as persistent homology, to confirm that the fundamental topology of these attribute spaces is equivalent across subjects (90–92). TDA has proven effective at revealing the structure of neural manifolds (93–96). To probe finer geometric details, the Gromov-Wasserstein Optimal Transport (GWOT) framework (97) is ideal. Unlike standard methods like Representational Similarity Analysis (RSA) (98), GWOT can compare the geometry of two spaces without requiring a predefined point-to-point correspondence, a major advantage when comparing different brains (24, 99–101). A strong correspondence in this stage would provide powerful evidence for a universal structure in subjective experience.

### Stage 2: the plastic structure of qualia signatures

This stage is an exploratory analysis of the plastic, learned geometry of qualia signatures (Fig. 6B). Since our theory predicts this structure is idiosyncratic, the goal is not to prove equivalence but to empirically quantify the degree of similarity between individuals. Here, methods sensitive to metric structure like RSA (98) and other neural alignment techniques (102) are well-suited. Furthermore, GWOT is again particularly useful, as it can determine whether any alignment between two individuals’ conceptual spaces is fine-grained or occurs only at a coarser, categorical level (101, 103).

This two-stage protocol thus serves as a nuanced diagnostic tool. A successful alignment in stage 1 would validate our framework’s core assumption of equivariant processing. The degree of alignment found in stage 2, which is not theoretically guaranteed, would then provide a quantitative measure of how shared pressures, such as language or culture, may guide different individuals to converge on similar conceptual structures.

## Approximate and emergent equivariance

Although the propositions derived from principal bundle geometry may appear self-evident in a purely mathematical context, they make powerful, empirically testable claims about the structure of any system that learns equivariant representations. This formulation provides more than just a descriptive theory; it offers a concrete research program for discovering nontrivial properties and achieving a deeper understanding of the qualia structure of a given neural system. For instance, as we proposed in the previous section, assessing individual differences in terms of orbits and quotient spaces is one such experimental approach to pursue in the future. The core of this program involves systematically applying transformations from a symmetry group *G* to sensory inputs and measuring the resulting changes in the neural representation space. Through this process, one can empirically map the geometry of the orbits and the quotient space.

However, the theoretical framework, which assumes perfect *G*-equivariance, is an idealization. In reality, any neural system will at best exhibit approximate equivariance. In artificial neural networks, achieving high degrees of equivariance often requires explicit engineering, such as specialized architectures, data augmentation, and loss functions designed to minimize equivariance error. In contrast, it is neurobiologically implausible that biological systems use such explicit optimization for a specific mathematical symmetry. Instead, any equivariance in the brain must be an emergent property of learning from noisy and imperfect real-world data under biological constraints, such as metabolic costs and neuronal wiring costs.

This reality shifts the focus toward a crucial set of empirical questions: What types of equivariance actually emerge in these systems, and how strongly are they expressed? Answering these questions requires robust methods for quantifying the deviation from perfect equivariance.

In artificial networks, several metrics have been developed for this purpose. These include the G-empirical equivariance deviation (G-EED) (104), which directly quantifies the extent to which a model’s output deviates from perfect equivariance, and the Local Equivariance Error (LEE) (105), which is derived from the Lie derivative and connects local deviations to global equivariance properties (see Ref. (106) for a unified perspective). Building on this Lie-derivative perspective, deeper integration of Lie algebra structure into the theoretical framework, including the geometry of approximate orbits and quotient spaces, is a natural future direction. Beyond simply measuring the degree of equivariance for known symmetries, such as rotation or translation, active research programs are also working to discover hidden or unknown symmetries directly from data, independent of prior knowledge (107–109).

Extending analogous measurements to the brain is more challenging because its input–output mapping can only be sampled indirectly. Nevertheless, a recent study introduced an in vivo framework that directly estimates local equivariance from large-scale neuronal recordings, revealing that cortical populations can interpolate between finely tuned and transformation-invariant codes (78). This approach provides a concrete route to quantifying equivariance in biological circuits and establishes a natural benchmark for comparing the operational symmetries of brains with those of artificial systems.

In practice, the framework’s empirical application proceeds bidirectionally and iteratively rather than presupposing that the neural correlate of a given experience is already established. Phenomenologically motivated predictions about an experience’s equivariance and orbit/quotient structure place geometric constraints on candidate neural substrates and indicate what structure to look for; conversely, empirical neural findings refine the phenomenological model in turn. Iterating between phenomenology and neural substrate, with each side constraining and refining the other, is how the framework supports concrete empirical progress on the structure of qualia and their neural correlates. This iterative methodology naturally extends to perceptual modalities, for example olfaction or gustation, whose phenomenal quality space is poorly mapped despite comparatively well-characterized neural circuitry, and where the framework’s geometric lens may help infer quality-space organization from neural data.

## Compositionality and hierarchical structures of qualia

**Figure 7:**
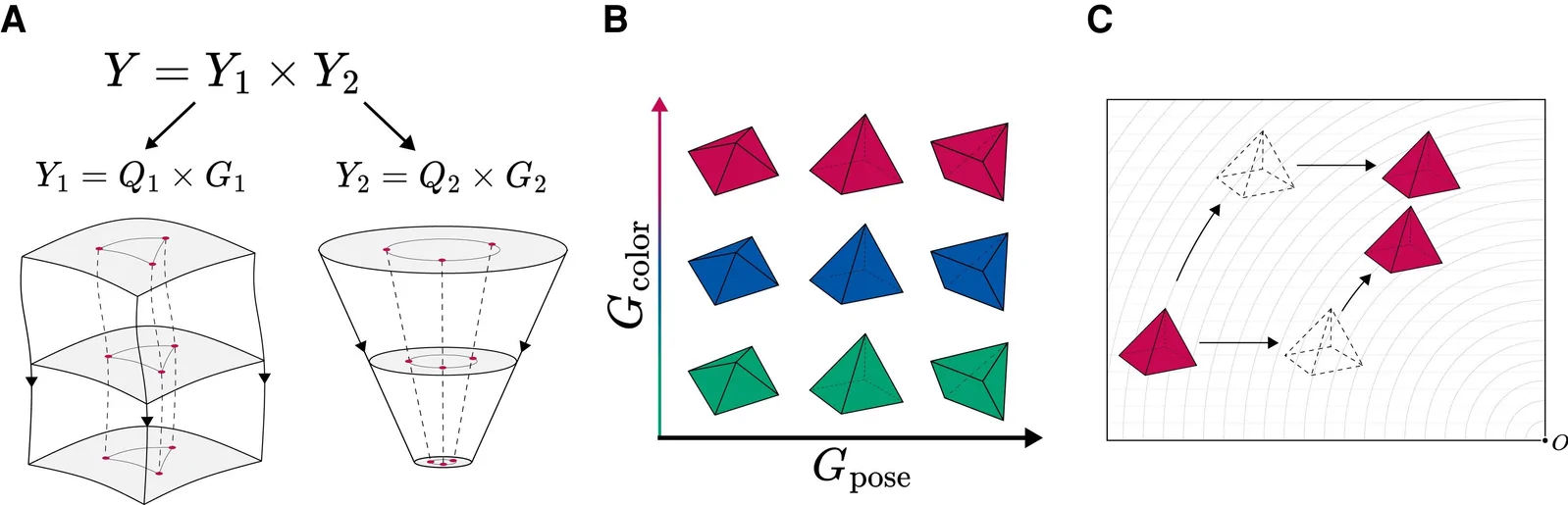
Compositionality and disentanglement of qualia. A) The decomposition of *Y* under disentangled symmetry group $G=G_{1}\times G_{2}$. Each factor space $Y_{1}$ and $Y_{2}$ is structured as a bundle over its respective quotient space. Different bundles encode distinct aspects of qualia, which are represented by qualia signatures in the quotient spaces, as well as by corresponding qualia attributes represented by orbits (dashed lines). B) A conceptual example of a disentangled representation where color and pose vary independently along orthogonal axes. This reflects a direct product symmetry group $G_{\mathrm{color}}\times G_{\mathrm{pose}}$, where each axis corresponds to transformations under a distinct subgroup action. C) Illustration of noncommutativity in the semidirect product $SE(2)=R^{2}⋊SO(2)$. Rotating the red tetrahedron (initially at lower left) around the frame’s lower-right corner (indicated by *O*) and then translating it right yields a different result than translating first and then rotating.

While the discussion so far has centered on single symmetry groups, conscious experience is generally compositional, involving multiple interacting symmetries. Our framework can be extended to model this complexity using fundamental algebraic structures that describe how different qualia attributes combine (see Supplementary material for mathematical details). For perceptually independent features, such as an object’s color and its pose, the total symmetry can be modeled as a direct product of groups ($G_{\mathrm{total}}=G_{1}\times G_{2}$). As illustrated in Fig. 7A, this structure geometrically corresponds to a factorization of the total experience into independent components. This means a neural system can, in principle, learn to represent different attributes along separate, orthogonal axes, as shown for color and pose in Fig. 7B. This concept of factorization is a central goal in the field of disentangled representation learning in AI (110–114), though achieving perfect disentanglement remains a significant challenge (30, 115).

On the other hand, a unified percept often requires a tighter, hierarchical integration of features where attributes are entangled. This is modeled by the semidirect product ($G_{\mathrm{total}}=G_{1}{⋊}_{\phi }G_{2}$), which describes a noncommutative relationship where the order of operations matters. Algebraically, this structure means that $G_{1}$ is a normal subgroup of $G_{\mathrm{total}}$ (i.e. $gG_{1}g^{-1}=G_{1}$ for all $g\in G_{\mathrm{total}}$), while $G_{2}$ acts on $G_{1}$ by automorphisms via *ϕ*. A classic example is the combination of translation ($G_{1}=R^{2}$) and rotation ($G_{2}=SO(2)$), which forms the special Euclidean group SE(2). As shown in Fig. 7C, translating an object and then rotating it yields a different outcome than rotating it first and then translating. This structure reflects how, perceptually, attributes like position and orientation are often entangled; an object’s orientation can change its effective position relative to a reference point. Geometrically, this corresponds to a hierarchical, composite bundle structure (116). Concretely, because $G_{1}$ is normal, the bundle $Y\to Y/G_{\mathrm{total}}$ factors as a staged quotient $Y\to Y/G_{1}\to (Y/G_{1})/G_{2}≅Y/G_{\mathrm{total}}$: at the first stage, $G_{1}$-orbits in *Y* are attributes and points in $Y/G_{1}$ are intermediate signatures; at the second stage, the induced $G_{2}$-action on $Y/G_{1}$ produces further orbits and a final quotient. The signature/attribute distinction therefore refines hierarchically with the chosen level of group action. The duality of rigid attributes and plastic signatures refines along the same hierarchy: at each stage, attributes are universal, while intermediate signatures carry residual rigid structure from the remaining group action. Irreducible plasticity resides only in the final quotient $Y/G_{\mathrm{total}}$. More generally, classical results on the factorization of groups into minimal components, such as direct and semidirect products and composition series, suggest a research direction in which such irreducible factors may serve as candidates for structural building blocks of perceptual organization. Together, these algebraic structures provide a rich mathematical language to model how the brain can both separate independent features and weave dependent ones into a single, coherent conscious experience.

## Conclusion and discussion

In this study, we proposed that the relational structure of qualia can be rigorously described by principal bundle geometry. To restate the scope clearly, the present framework is a structural theory of perceptual qualia in systems that we have independent reason to regard as conscious, not a criterion for consciousness as such. Our central thesis is that when a neural system learns to process sensory information in a way that is equivariant to a symmetry group *G* realized by the neural encoder, its internal state space naturally acquires this geometric structure. This provides a three-level characterization of experience: the qualia modality is constrained by the group *G* itself, the qualia signature by a point in the quotient space *Q*, and the qualia attribute by a point on an orbit O. The core consequence of this framework is the duality of “rigid attributes and plastic signatures.” It explains how perceptual systems can maintain a stable, universal relational structure for continuous attributes (e.g. location, hue) while developing flexible, idiosyncratic geometric representations for invariant signatures (e.g. object identity). This theoretical lens opens up several new avenues for empirical research.

The immediate priority is the empirical validation of our framework’s predictions. The proposed two-stage testing protocol is crucial for this, using methods like TDA and GWOT to first verify the predicted universal structure of qualia attributes (orbits). The exploratory second stage can then assess the degree of similarity in the geometric structure of qualia signatures (quotient spaces) across individuals. While our theory posits this signature space is plastic, its geometry may not be entirely idiosyncratic. We hypothesize that shared pressures, particularly language, could drive a convergence of these learned structures across people. This aligns with modern interpretations of linguistic relativity (117) and evidence that language acts as a scaffold for shared meaning (118, 119), potentially aligning the semantic geometry of individuals (120), much like in large-scale vision-language models (121). Such alignment would explain the remarkable consistency observed in neural representations of object categories across individuals (122, 123).

Beyond higher-level pressures like language, a more fundamental constraint imposed by our framework may explain the observed consistency in qualia structures. A key empirical question is just how vast the total symmetry group G is that the brain learns. We must be equivariant not to one or two simple transformations, but to a rich compositional group reflecting the myriad symmetries of the physical world—translations, rotations, scaling, lighting changes, and countless others. Given the brain’s finite representational capacity, dedicating a large portion of its neural geometry to forming these numerous, rigid orbit structures necessarily leaves a smaller and more constrained quotient space. In this view, the “plastic” space of qualia signatures is not a vast, unconstrained canvas but is instead a limited territory, heavily shaped by the sheer dominance of the universal geometry it is built upon. This powerful physical-geometric constraint itself, even prior to any cultural or linguistic influence, may be a primary reason why our subjective worlds are so fundamentally alike.

Our framework is not itself a general theory of consciousness; rather, it is a structural characterization of the geometry of perceptual qualia in candidate conscious systems. Within this restricted scope, it is nonetheless instructive to compare the geometric structure that our framework predicts with the structures posited by leading theories of consciousness (16, 17). Concerning the attempt to understand the nature of qualia from geometric perspectives, our framework’s most salient contrast is with Integrated Information Theory (IIT), a pioneering research program that also connects physical structure to the quality of experience (124–126). The critical difference lies in the source of this structure. Our theory explains the relational structure of qualia as the geometry induced by G-equivariant feed-forward functions that learn and preserve extrinsic symmetries from the input space. In contrast, IIT posits that consciousness is identical to the cause-effect structure arising from the intrinsic causal interactions of recurrent networks. This divergence is clear in their respective accounts of spatial experience. For IIT, the extended feeling of space arises from the grid-like architecture of intrinsic neural connections (127). For our framework, it is inherited from processing external symmetry groups like translation and rotation. Clarifying the relationship between IIT”s intrinsic causal structures and our framework’s group-induced geometric structures remains an important open question.

In conclusion, applying principal bundle geometry to the study of qualia offers a rich, mathematically grounded, and empirically testable approach. By focusing on symmetry, it provides a unifying language that connects group theory, neural population activity, and the phenomenology of subjective experience, holding significant promise for understanding the structural properties of perceptual qualia from their physical substrate.

## Supplementary Material

pgag261_Supplementary_Data

## Data Availability

This article is a theoretical study and does not generate or analyze new datasets or computer code. All conceptual content is contained within the article and its Supplementary material.

## Preprints

This manuscript was posted as a preprint at https://doi.org/10.31234/osf.io/agupq_v3.
