# Correction to: Principal bundle geometry of qualia: Understanding the quality of consciousness from symmetry

**DOI:** 10.1093/pnasnexus/pgag313

**Published:** 2026-09-29

This is a correction to: Masafumi Oizumi, Chanseok Lim, Ryota Kanai, Principal bundle geometry of qualia: Understanding the quality of consciousness from symmetry, PNAS Nexus, Volume 5, Issue 9, September 2026, pgag261, https://doi.org/10.1093/pnasnexus/pgag261

Due to a production error, an unrelated Supplementary material file was originally published with this paper.

This error has been corrected, and the article has been updated with the correct Supplementary material. In addition, Figure 4 has been re-oriented. The panels have been arranged horizontally so that the image is "right-side up".

