# Supplementary material for: Principal bundle geometry of qualia: Understanding the quality of consciousness from symmetry
Source: PNAS Nexus. 2026 Sep 8;5(9):pgag261. doi: 10.1093/pnasnexus/pgag261 (PMC13552501; doi:10.1093/pnasnexus/pgag261)
Supplement: Supplementary file 1 [file pgag261_supplementary_data.pdf]

Supplementary Material

Principal bundle geometry of qualia: Understanding the quality of consciousness from symmetry

Masafumi Oizumi, Chanseok Lim, and Ryota Kanai

Contents

- Definition of a principal bundle
- Propositions for rigidity of orbits and plasticity of quotient space
- Proofs of propositions
- Compositionality and hierarchical structures of qualia

Definition of a principal bundle

In the main text, we propose that the geometry of qualia is characterized by a principal bundle. Here, we provide a formal definition of this mathematical structure.

A principal bundle consists of a total space Y, a base space Q, a Lie group G, and a continuous surjective map $\pi : Y \to Q$ that satisfies the following conditions:

1. **Group action:** The group G acts on the total space Y from the right. This action is smooth and free, meaning that for any point $y \in Y$, if $y \cdot g = y$ for some $g \in G$, then g must be the identity element of the group.
2. **Projection and orbits:** The projection map π is constant on the orbits of the group action. This means that for any point $y \in Y$ and any group element $g \in G$, $\pi(y \cdot g) = \pi(y)$. Consequently, the base space Q is the quotient space of Y by the group action, written as $Q = Y/G$. The pre-image $\pi^{-1}(q)$ for any point $q \in Q$ is an orbit of the group action and is called a fiber.
3. **Local triviality:** The bundle is locally a product space. For any point $q \in Q$, there exists an open neighborhood U of q in the base space such that the pre-image $\pi^{-1}(U)$ is homeomorphic to the Cartesian product $U \times G$. This homeomorphism, $\phi : \pi^{-1}(U) \to U \times G$, is required to be equivariant in the sense that $\phi(y \cdot g) = (\pi(y), \phi_G(y) \cdot g)$, where $\phi_G(y)$ is the group component of $\phi(y)$. This condition ensures that the structure of the fibers is locally consistent and identical to the structure of the group G itself.

In the context of our theory, the neural representation space Y is the total space, the symmetry group G defines the qualia modality, the orbits (fibers) represent qualia attributes, and the quotient space (base space) Q represents qualia signatures.

Propositions for rigidity of orbits and plasticity of quotient space

In this section, we develop the geometric implications of our framework. Our central claim is that the principal bundle geometry induced by the symmetry group imposes a powerful duality on the structure of qualia: the geometry of qualia attributes is topologically rigid and universal, while the geometry of qualia signatures is plastic and learnable. We first formalize the conditions for this structure to arise. Then, we mathematically prove the rigidity of the orbits, representing qualia attributes, and the plasticity of the quotient space, representing qualia signatures.

We will now establish this duality through a series of propositions. Propositions S1–S4 will establish the rigidity of the orbits, showing that they are topologically identical across subjects and can be metrically rigid under stronger conditions. In contrast, Propositions S5–S7 will address the plasticity of the quotient space, demonstrating that its geometry can be learned and vary across subjects, while remaining coherent from different viewpoints within a subject.

In the following, we assume that the symmetry group G is a Lie group [1] acting smoothly, properly, and freely on an input space X. The neural representation space Y is a d-dimensional Euclidean space, $Y \simeq \mathbb{R}^d$. The group acts freely on Y via a continuous linear representation $\rho\colon G \to GL(d)$. The neural encoder $f\colon X \to Y$ is assumed to be exactly equivariant, satisfying $f(g \cdot x) = \rho(g) f(x)$.

Rigidity of orbits: The universal structure of qualia attributes

The equivariance of the encoder f has profound consequences for the geometry of the orbits. It ensures that their fundamental topological structure is not learned or arbitrary but is rigidly inherited from the symmetry group G itself. This rigidity can be formalized in the following propositions. Their proofs are provided in the section “Proofs of propositions” below.

Proposition S1 (Orbit-group diffeomorphism; Figure 5A in the main text). *Let $f : X \to Y$ be a smooth, proper, G-equivariant map between smooth G-spaces X and Y. Assume the action on X is free and proper. Then for any $x_0 \in X$, if the action of G on Y is free at $y_0 = f(x_0)$, the orbit $\mathcal{O}_Y(y_0)$ is a properly embedded smooth submanifold of Y that is diffeomorphic to G.*

This proposition establishes a strong topological rigidity. The general result of topological rigidity leads to a powerful conclusion about comparing qualia across different individuals or networks.

Proposition S2 (Inter-subject orbit diffeomorphism; Figure 5A in the main text). *Let Person A have an equivariant encoder $f_A : X \to Y_A$ (with representation ρ_A) and Person B have an equivariant encoder $f_B : X \to Y_B$ (with representation ρ_B), both with respect to the same Lie group G. Let $x_0 \in X$ be a point in the stimulus space, and let $y_A = f_A(x_0) \in Y_A$ and $y_B = f_B(x_0) \in Y_B$ be the corresponding neural representations. If the G-actions on Y_A and Y_B are free at y_A and y_B respectively, then the orbit $\mathcal{O}_{Y_A}(y_A)$ is diffeomorphic to the orbit $\mathcal{O}_{Y_B}(y_B)$*

A stricter, metric rigidity is achieved under the additional condition of orthogonality. When the representation is orthogonal, the orbit inherits a canonical metric geometry from the group G itself. This means that the geometry of the group space is homogeneous, that is, the distance between any two transformations g_1 and g_2 depends only on the relative transformation $g_2^{-1} g_1$ needed to get from one to the other, not on their absolute position within the group. This provides a rigid, canonical “ruler” for measuring distances on the orbit. The following proposition makes this precise.

Proposition S3 (Orbit-group isometry)**.** *Suppose the neural representation space Y is endowed with a Riemannian metric g_Y (for example, the Euclidean metric). If the representation $\rho : G \to O(d)$ is an orthogonal representation, then for any $y_0 \in Y$ in the image of f, the orbit $\mathcal{O}_Y(y_0)$, endowed with the induced Riemannian metric, is isometric to the Lie group G, endowed with the pullback Riemannian metric $\phi_Y^* g_Y$. Furthermore, this pullback metric $\phi_Y^* g_Y$ on G is a left-invariant Riemannian metric.*

By the assumption of orthogonal representation, there is a much stronger similarity between the orbits across different individuals than a mere diffeomorphism. The orthogonality condition ensures that each orbit inherits a rigid, homogeneous geometry from the group itself, where the geometry at any point is a simple translation of the geometry at the identity. Consequently, the diffeomorphism Ψ connecting two such orbits cannot be an arbitrary local warping. Instead, the distortion required to map one orbit onto the other must be globally uniform, dictated by a single linear transformation on the underlying Lie algebra. In the special case where this transformation is a uniform scaling, the orbits are related by a homothety. If this scaling factor is one, the orbits are metrically identical (isometry). This leads to the following proposition.

Proposition S4 (Uniform metric distortion of inter-subject orbit maps)**.** *Let $\mathcal{O}_{Y_A}(y_A)$ and $\mathcal{O}_{Y_B}(y_B)$ be two orbits generated by orthogonal representations. The entire geometric relationship between them is determined by a single, constant, self-adjoint linear operator $L : \mathfrak{g} \to \mathfrak{g}$ on the Lie algebra $\mathfrak{g}$, which relates the two pullback metrics at the identity: $\langle X, Z\rangle_B = \langle X, L(Z)\rangle_A$. In the special case where this operator is a scalar multiple of the identity, $L = c \cdot \mathrm{Id}$, the map Ψ is a homothety.*

Consequence for qualia attributes. These propositions provide a formal basis for the rigidity of qualia attributes. The fundamental topological structure of how attributes relate to one another (e.g., that hues are arranged in a circle) is a universal consequence of the underlying symmetry group G, as guaranteed by Proposition S1. This topological equivalence is universal across individuals; even if two subjects learn very different neural representations ($\rho_A \neq \rho_B$), Proposition S2 implies that the structure of their corresponding qualia attributes must be diffeomorphic. This constrains individual differences in experience to be, at most, smooth warpings of a universal geometric object.

A much stricter, metric rigidity is addressed in the subsequent propositions. If a neural representation is orthogonal, the orbit's intrinsic geometry becomes a rigid, homogeneous copy of the group G itself, endowed with a canonical left-invariant metric (Proposition S3). Because both subjects' orbits are then metrically rigid copies of the same group, the mapping Ψ that connects their qualia spaces cannot be an arbitrary warping. Instead, as described in Proposition S4, the distortion must be globally uniform, dictated by a single, constant linear operator on the underlying Lie algebra. In the special case where this operator is a simple scaling, the map is a homothety, meaning the structure of the two qualia attributes has the exact same shape.

Plasticity of quotient space: The learnable structure of qualia signatures

While the orbits that define qualia attributes have a rigid topological structure, the geometry between orbits, which defines the relationships between qualia signatures, is plastic. This plasticity allows a neural system to flexibly organize its conceptual space based on learning and task demands.

To analyze the geometry of the quotient space Q_Y, it is useful to have a concrete representation of this space within the larger neural manifold Y. Such a representation is given by a section, which is a submanifold $S \subset Y$ that intersects each orbit exactly once. For the sake of a clear geometric analysis, we will assume that a smooth global section exists. This assumption simplifies the picture by ensuring that the neural representation space Y is not globally twisted, a condition mathematically equivalent to the principal bundle being trivial, i.e., globally diffeomorphic to a product space $Y \cong Q_Y \times G$ (see, e.g. [2]). The subsequent propositions explore the geometry of this section S.

Proposition S5 (Quotient plasticity; Figure 5B in the main text)**.** *Let S be a smooth global section of Y corresponding to a G-equivariant encoder f. The intrinsic geometry of S is not fixed by the G-equivariance constraint alone. By composing the encoder f with a valid G-equivariant linear map, the geometry of the resulting section $\tilde{S}$ can be arbitrarily altered.*

This plasticity explains why different individuals can learn and refine their understanding of the world in different ways, depending on task demands or experiences. This leads immediately to the next proposition, which states that the quotient space structure is person-specific:

Proposition S6 (Inter-subject quotient plasticity; Figure 5B in the main text)**.** *Let Person A and Person B have encoders f_A and f_B that are both equivariant with respect to the same group G. Let S_A and S_B be any corresponding sections of Y_A and Y_B, respectively. There is no guaranteed geometric relationship (e.g., isometry, similarity, or even diffeomorphism) between the submanifolds S_A and S_B with their inherited Riemannian metrics.*

Finally, although the geometry of the quotient space is learnable, we remark that it must be coherent across different viewpoints for a single individual. This ensures that the structure of the space of signatures remains consistent, regardless of the specific attributes (e.g., location, orientation) through which they are viewed. This intra-subject coherence is formalized by the following proposition.

Proposition S7 (Intra-subject quotient diffeomorphism)**.** *Let $s_1 : Q_Y \to Y$ be a smooth global section, and let $S_1 = s_1(Q_Y)$ be its image. For any group element $g \in G$, let S_2 be the image of the section $s_2 = \rho(g) \circ s_1$. The map $\Psi_g : S_1 \to S_2$ defined by restricting the group action, $\Psi_g(y) = \rho(g)y$, is a diffeomorphism.*

Consequence for qualia signatures. These propositions provide a formal basis for the plasticity of qualia signatures. Proposition S5 shows why different individuals can refine their internal structure of qualia signatures in different ways. However, note that Proposition S7 ensures that this learned

map is internally coherent and stable across viewpoints within the same individual. It shows that the geometric structure of the quotient space is consistent regardless of the specific qualia attributes (e.g., location or orientation) through which the signatures are viewed. Finally, Proposition S6 immediately follows from Proposition S5, formalizing the idea that this coherent, learned structure is not universal but idiosyncratic. Two people can learn to structure qualia signatures in radically different ways, depending on their experiences, goals, and even random variations in their neural wiring, all while sharing a universal, topologically rigid structure for the attributes within each of those signatures. This captures the duality of a shared physical world and a private, learned understanding of it (Figure 5A and B in the main text).

Proofs of propositions

Rigidity of orbits

Proposition S1 (Orbit-group diffeomorphism; Figure 5A in the main text)**.** *Let $f : X \to Y$ be a smooth, proper, G-equivariant map between smooth G-spaces X and Y. Assume the action on X is free and proper. Then for any $x_0 \in X$, if the action of G on Y is free at $y_0 = f(x_0)$, the orbit $\mathcal{O}_Y(y_0)$ is a properly embedded smooth submanifold of Y that is diffeomorphic to G.*

Proof. Let $y_0 \in \mathrm{Im}(f)$, and choose a point $x_0 \in X$ such that $f(x_0) = y_0$. The proof demonstrates that the orbit map from G to the orbit of y_0 is a proper embedding, which establishes the result.

The orbit map is a smooth injective immersion. The orbit of y_0 is the image of the orbit map $\phi_{y_0} : G \to Y$, defined by $\phi_{y_0}(g) = \rho_Y(g)y_0$. The map ϕ_{y_0} is smooth because it is the composition of the smooth map $g \mapsto (g, y_0)$ and the smooth action map $G \times Y \to Y$.

We are given that the action on Y is free at y_0, meaning the isotropy group $G_{y_0} = \{g \in G \mid \rho_Y(g)y_0 = y_0\}$ is trivial. This implies ϕ_{y_0} is injective.

By Proposition 7.26 [3], every orbit map of a smooth Lie group action has constant rank. Since ϕ_{y_0} is an injective smooth map of constant rank, the Global Rank Theorem (Theorem 4.14(b) [3]) implies it is a smooth immersion.

The orbit map is a proper map. A map is proper if the preimage of every compact set is compact. The equivariance of f is used here to relate the actions on X and Y.

- The G-equivariance of f establishes the identity $\phi_{y_0} = f \circ \phi_{x_0}$, where $\phi_{x_0}(g) = \rho_X(g)x_0$.
- We are given that the action of G on X is proper. By Proposition 21.7 [3], this implies that the orbit map $\phi_{x_0} : G \to X$ is a proper map.
- We are also given that the map $f : X \to Y$ is a proper map.
- The composition of two proper maps is proper. Therefore, the map $\phi_{y_0} = f \circ \phi_{x_0}$ is a proper map from G to Y.

Conclusion. We have shown that $\phi_{y_0} : G \to Y$ is a proper, injective, smooth immersion.

- By Proposition 4.22(b) [3], an injective proper immersion is a smooth embedding.
- The image of a smooth embedding is an embedded submanifold (Proposition 5.2 [3]). As the image of ϕ_{y_0} is the orbit $\mathcal{O}_Y(y_0)$, the orbit is an embedded submanifold of Y.
- Because ϕ_{y_0} is a proper map and Y is a locally compact Hausdorff space, its image $\mathcal{O}_Y(y_0)$ is a closed subset of Y (Theorem A.57 [3]). An embedded submanifold that is also a closed subset is a properly embedded submanifold (Proposition 5.5 [3]).
- Finally, since ϕ_{y_0} is a smooth embedding, it is a diffeomorphism from its domain G onto its image $\mathcal{O}_Y(y_0)$.

Thus, the orbit $\mathcal{O}_Y(y_0)$ is a properly embedded smooth submanifold of Y that is diffeomorphic to the Lie group G. □

Proposition S2 (Inter-subject orbit diffeomorphism; Figure 5A in the main text)**.** *Let Person A have an equivariant encoder $f_A : X \to Y_A$ (with representation ρ_A) and Person B have an equivariant encoder $f_B : X \to Y_B$ (with representation ρ_B), both with respect to the same Lie group G. Let $x_0 \in X$ be a point in the stimulus space, and let $y_A = f_A(x_0) \in Y_A$ and $y_B = f_B(x_0) \in Y_B$ be the corresponding neural representations. If the G-actions on Y_A and Y_B are free at y_A and y_B respectively, then the orbit $\mathcal{O}_{Y_A}(y_A)$ is diffeomorphic to the orbit $\mathcal{O}_{Y_B}(y_B)$*

Proof. The objective is to demonstrate that the orbits $\mathcal{O}_{Y_A}(y_A)$ and $\mathcal{O}_{Y_B}(y_B)$ are diffeomorphic. This proof hinges on the principle that if two spaces are each diffeomorphic to a third common space, then they are diffeomorphic to each other. In this context, the common third space is the Lie group G.

1. **Application of Proposition S1:** Proposition S1 states that for a G-equivariant system where the Lie group G acts smoothly, freely, and properly on a smooth manifold Y, any orbit $\mathcal{O}_Y(y_0)$ (for $y_0 \in Y$ in the image of the encoder) is a smooth submanifold of Y that is diffeomorphic to G. The orbit map $\phi_Y : G \to \mathcal{O}_Y(y_0)$, defined by $\phi_Y(g) = \rho(g)y_0$, is the diffeomorphism that establishes this relationship.
   - For Person A's system: Given the anchor point $y_A = f_A(x_0)$, and assuming the G-action on Y_A is free at y_A, Proposition S1 ensures the existence of a diffeomorphism $\phi_A : G \to \mathcal{O}_{Y_A}(y_A)$.
   - For Person B's system: Similarly, with the anchor point $y_B = f_B(x_0)$, and given that the G-action on Y_B is free at y_B, Proposition S1 implies the existence of a diffeomorphism $\phi_B : G \to \mathcal{O}_{Y_B}(y_B)$.
2. **Construction of the inter-subject diffeomorphism:** Since both $\mathcal{O}_{Y_A}(y_A)$ and $\mathcal{O}_{Y_B}(y_B)$ are diffeomorphic to the same Lie group G, it follows that $\mathcal{O}_{Y_A}(y_A)$ is diffeomorphic to $\mathcal{O}_{Y_B}(y_B)$. An explicit diffeomorphism $\Psi : \mathcal{O}_{Y_A}(y_A) \to \mathcal{O}_{Y_B}(y_B)$ can be constructed by composing ϕ_B with the inverse of ϕ_A, i.e., $\Psi = \phi_B \circ \phi_A^{-1}$. A diffeomorphism is defined as a bijective smooth map with a smooth inverse. The inverse of a diffeomorphism is itself a diffeomorphism, and the composition of two diffeomorphisms is also a diffeomorphism. Therefore, Ψ is indeed a diffeomorphism, completing the proof.

□

Proposition S3 (Orbit-group isometry)**.** *Suppose the neural representation space Y is endowed with a Riemannian metric g_Y (for example, the Euclidean metric). If the representation*

$\rho : G \to O(d)$ is an orthogonal representation, then for any $y_0 \in Y$ in the image of f, the orbit $\mathcal{O}_Y(y_0)$, endowed with the induced Riemannian metric, is isometric to the Lie group G, endowed with the pullback Riemannian metric $\phi_Y^* g_Y$. Furthermore, this pullback metric $\phi_Y^* g_Y$ on G is a left-invariant Riemannian metric.

Proof. Let g_Y be the Riemannian metric on the ambient space Y. The orbit $\mathcal{O}_Y(y_0)$ inherits an induced Riemannian metric from Y, which we also denote by g_Y. Let g_G denote the pullback metric on the Lie group G, defined by $g_G = \phi_{y_0}^* g_Y$, where $\phi_{y_0} : G \to \mathcal{O}_Y(y_0)$ is the orbit map defined by $\phi_{y_0}(g) = \rho(g)y_0$.

1. The orbit-group map ϕ_{y_0} is an isometry. An isometry is a diffeomorphism that preserves the Riemannian metric. From Proposition S1, we know that the orbit map $\phi_{y_0} : G \to \mathcal{O}_Y(y_0)$ is a diffeomorphism. We must show it preserves the metric. For any tangent vectors $X, Z \in T_gG$ at a point $g \in G$, the definition of the pullback metric is:

$$(g_G)_g(X, Z) := (g_Y)_{\phi_{y_0}(g)}(d(\phi_{y_0})_g(X), d(\phi_{y_0})_g(Z)) \tag{S1}$$

This equation states that the inner product of two vectors in G (with metric g_G) is defined to be the inner product of their images under the differential $d\phi_{y_0}$ in the orbit (with metric g_Y). By its very construction, the map $\phi_{y_0} : (G, g_G) \to (\mathcal{O}_Y(y_0), g_Y)$ is an isometry.

2. The pullback metric g_G is left-invariant. This is the main result, and it relies critically on the representation ρ being orthogonal. A metric g_G is left-invariant if, for every $h \in G$, the left-translation map $L_h : G \to G$ is an isometry, i.e., $L_h^* g_G = g_G$. We evaluate the pullback of g_G by L_h at an arbitrary point $g \in G$ on arbitrary tangent vectors $X, Z \in T_gG$:

$$\begin{aligned}(L_h^* g_G)_g(X, Z) &= (g_G)_{L_h(g)}(d(L_h)_g(X), d(L_h)_g(Z)) \\ &= (g_G)_{hg}(d(L_h)_g(X), d(L_h)_g(Z))\end{aligned} \tag{S2}$$

Now, we substitute the definition of $g_G = \phi_{y_0}^* g_Y$:

$$\begin{aligned}&= (g_Y)_{\phi_{y_0}(hg)}\Big(d(\phi_{y_0})_{hg}\big(d(L_h)_g(X)\big), \\ &\qquad d(\phi_{y_0})_{hg}\big(d(L_h)_g(Z)\big)\Big)\end{aligned} \tag{S3}$$

Using the chain rule, $d(\phi_{y_0})_{hg} \circ d(L_h)_g = d(\phi_{y_0} \circ L_h)_g$. From the group action, we have the relation $\phi_{y_0}(hg) = \rho(h)(\phi_{y_0}(g))$, which means $\phi_{y_0} \circ L_h = \rho(h) \circ \phi_{y_0}$. Taking the differential at g:

$$d(\phi_{y_0} \circ L_h)_g = d(\rho(h) \circ \phi_{y_0})_g = d(\rho(h))_{\phi_{y_0}(g)} \circ d(\phi_{y_0})_g \tag{S4}$$

Since $\rho(h)$ is a linear map, its differential is just itself. So, $d(\phi_{y_0} \circ L_h)_g = \rho(h) \circ d(\phi_{y_0})_g$. Substituting this back into our main expression gives:

$$\begin{aligned}(L_h^* g_G)_g(X, Z) = (g_Y)_{\rho(h)(\phi_{y_0}(g))}\Big(&(\rho(h)(d(\phi_{y_0})_g(X)), \\ &\rho(h)(d(\phi_{y_0})_g(Z))\Big)\end{aligned} \tag{S5}$$

This is where we use the crucial assumption that ρ is an **orthogonal representation**. By definition, this means that for any $h \in G$, the linear map $\rho(h)$ is an orthogonal transformation and preserves the inner product g_Y. Therefore:

$$(g_Y)_{\rho(h)(\phi_{y_0}(g))}(\rho(h)(v_1), \rho(h)(v_2)) = (g_Y)_{\phi_{y_0}(g)}(v_1, v_2) \tag{S6}$$

Applying this property, our expression simplifies to:

$$(L_h^* g_G)_g(X, Z) = (g_Y)_{\phi_{y_0}(g)}(d(\phi_{y_0})_g(X), d(\phi_{y_0})_g(Z)) \tag{S7}$$

By the definition of the pullback metric, this is exactly $(g_G)_g(X, Z)$. Since this holds for all g, X, Z, we have shown $L_h^* g_G = g_G$, which proves that the pullback metric is left-invariant. □

Proposition S4 (Uniform metric distortion of inter-subject orbit maps)**.** *Let $\mathcal{O}_{Y_A}(y_A)$ and $\mathcal{O}_{Y_B}(y_B)$ be two orbits generated by orthogonal representations. The entire geometric relationship between them is determined by a single, constant, self-adjoint linear operator $L : \mathfrak{g} \to \mathfrak{g}$ on the Lie algebra $\mathfrak{g}$, which relates the two pullback metrics at the identity: $\langle X, Z\rangle_B = \langle X, L(Z)\rangle_A$. In the special case where this operator is a scalar multiple of the identity, $L = c \cdot \mathrm{Id}$, the map Ψ is a homothety.*

Proof. The proof proceeds by first establishing that the geometry of each orbit is determined by a single inner product on the Lie algebra $\mathfrak{g}$. Then, it shows that the relationship between these two inner products is a constant linear operator, which in turn dictates the nature of the diffeomorphism Ψ between the orbits.

1. Left-invariant metrics on G. From Proposition S3, we know that because the representations $\rho_A : G \to O(d_A)$ and $\rho_B : G \to O(d_B)$ are orthogonal, the pullback metrics on the Lie group G, defined as $g_A = \phi_A^* g_{Y_A}$ and $g_B = \phi_B^* g_{Y_B}$, are both left-invariant Riemannian metrics. A left-invariant metric is uniquely determined by its value at the identity element $e \in G$. This value is an inner product on the Lie algebra $\mathfrak{g} = T_eG$. We denote these two inner products as $\langle \cdot, \cdot \rangle_A = (g_A)_e$ and $\langle \cdot, \cdot \rangle_B = (g_B)_e$.

2. The linear operator relating the geometries. We now have two distinct inner products, $\langle \cdot, \cdot \rangle_A$ and $\langle \cdot, \cdot \rangle_B$, defined on the same finite-dimensional vector space $\mathfrak{g}$. By a standard result from linear algebra, for any two such inner products, there exists a unique linear operator $L : \mathfrak{g} \to \mathfrak{g}$ which is self-adjoint with respect to $\langle \cdot, \cdot \rangle_A$ and relates the two inner products as follows for all $X, Z \in \mathfrak{g}$:

$$\langle X, Z\rangle_B = \langle X, L(Z)\rangle_A \tag{S8}$$

This single, constant linear operator L completely encapsulates the difference between the two metric geometries at the infinitesimal level of the Lie algebra.

3. From the Lie algebra to the orbits. The diffeomorphism between the two orbits is given by $\Psi = \phi_B \circ \phi_A^{-1}$. The geometric distortion induced by this map is characterized by the pullback metric $\Psi^* g_{Y_B}$. We can relate this back to the metrics on the group G:

$$\Psi^* g_{Y_B} = (\phi_B \circ \phi_A^{-1})^* g_{Y_B} = (\phi_A^{-1})^* (\phi_B^* g_{Y_B}) = (\phi_A^{-1})^* g_B \tag{S9}$$

Because both g_A and g_B are left-invariant, the relationship between them is uniform across the entire group. The linear

relationship L at the identity extends globally. That is, at any point $g \in G$ and for any left-invariant vector fields X, Y, we have $(g_B)_g(X, Y) = (g_A)_g(X, L(Y))$. This means the distortion is not local but uniform.

4. The special case of homothety. We now consider the special case where the operator L is a scalar multiple of the identity, i.e., $L = c \cdot \mathrm{Id}$ for some constant $c > 0$. In this case, the relationship between the inner products on the Lie algebra simplifies to:

$$\langle X, Z\rangle_B = \langle X, c \cdot Z\rangle_A = c\langle X, Z\rangle_A \tag{S10}$$

By left-invariance, this constant proportionality extends to the entire group, so $g_B = c \cdot g_A$. Substituting this into our pullback expression from paragraph 3:

$$\Psi^* g_{Y_B} = (\phi_A^{-1})^* g_B = (\phi_A^{-1})^*(c \cdot g_A) = c \cdot (\phi_A^{-1})^* g_A \tag{S11}$$

From Proposition S3, the map $\phi_A : (G, g_A) \to (\mathcal{O}_A, g_{Y_A})$ is an isometry. Therefore, its inverse ϕ_A^{-1} is also an isometry, and the pullback of g_A by ϕ_A^{-1} is the original induced metric on the orbit, g_{Y_A}.

$$(\phi_A^{-1})^* g_A = g_{Y_A} \tag{S12}$$

This yields the final result for the special case:

$$\Psi^* g_{Y_B} = c \cdot g_{Y_A} \tag{S13}$$

This is the definition of a homothety. □

Plasticity of quotient space

Proposition S5 (Quotient plasticity; Figure 5B in the main text)**.** *Let S be a smooth global section of Y corresponding to a G-equivariant encoder f. The intrinsic geometry of S is not fixed by the G-equivariance constraint alone. By composing the encoder f with a valid G-equivariant linear map, the geometry of the resulting section $\tilde{S}$ can be arbitrarily altered.*

Proof. The proposition asserts that the intrinsic metric geometry of a section (representing qualia signatures) is a plastic and learnable property of the neural representation. We prove this by demonstrating that a valid G-equivariant linear map can arbitrarily alter the geometry of a given section, a mechanism rooted in the nature of group representations.

1. **Initial setup:** Let $f : X \to Y$ be an initial G-equivariant encoder, and let $s : Q_Y \to Y$ be a corresponding smooth global section. Its image, $S = s(Q_Y)$, is an embedded submanifold of Y that inherits its intrinsic geometry (distances and angles) from the Euclidean metric of the ambient space Y.
2. **The role of G-equivariant linear maps:**
   - Consider a G-equivariant linear map $L : Y \to Y$. Such a map commutes with the group action, meaning $L \circ \rho(g) = \rho(g) \circ L$ for all $g \in G$, where ρ is the representation of G on Y.
   - If Person A modifies their encoder by composing it with such a map, say $L_A : Y_A \to Y_A$, they form a new valid encoder $\tilde{f}_A = L_A \circ f_A$. This transformation maps the original section S_A to a new submanifold $\tilde{S}_A = L_A(S_A)$, which serves as a valid section for the new encoder.
3. **Arbitrary alteration of geometry:** We now show that the intrinsic geometry of $\tilde{S}$ is generally different from that of S. Let y_1, y_2 be two distinct points within the original section S. The squared distance between them, which defines the metric on S, is $||y_1 - y_2||_2^2$.

   These points are mapped to $\tilde{y}_1 = L(y_1)$ and $\tilde{y}_2 = L(y_2)$ in the new section $\tilde{S}$. The squared distance between these new points is:

   $$||\tilde{y}_1 - \tilde{y}_2||_2^2 = ||L(y_1) - L(y_2)||_2^2 = ||L(y_1 - y_2)||_2^2.$$

   According to Schur's Lemma (see [4] for example), if the representation ρ is irreducible, any such commuting map L must be a scalar multiple of the identity, $L = \lambda I$, which would only scale the geometry uniformly. However, representations in neural systems are generally reducible. This means the representation space Y can be decomposed into a direct sum of irreducible subspaces, $Y = V_1 \oplus V_2 \oplus \cdots \oplus V_k$. The G-equivariant map L can then be a block-diagonal matrix, applying a different scaling factor λ_i to each subspace V_i.

   Unless the vector $(y_1 - y_2)$ lies entirely within a single irreducible subspace where $|\lambda_i| = 1$, the map L will change its length. Since vectors connecting points within a section are not generally constrained in this way, one can choose the scaling factors (λ_i) to arbitrarily expand, contract, or warp the distances within the section.

Therefore, the intrinsic geometry of a section is not fixed by G-equivariance alone, but can be arbitrarily altered by composing the encoder with a G-equivariant linear map, due to the reducibility of neural representations. □

Proposition S6 (Inter-subject quotient plasticity; Figure 5B in the main text)**.** *Let Person A and Person B have encoders f_A and f_B that are both equivariant with respect to the same group G. Let S_A and S_B be any corresponding sections of Y_A and Y_B, respectively. There is no guaranteed geometric relationship (e.g., isometry, similarity, or even diffeomorphism) between the submanifolds S_A and S_B with their inherited Riemannian metrics.*

Proof. The proposition states that no specific geometric relationship is guaranteed to hold between corresponding sections S_A and S_B from two different subjects. We prove this by demonstrating that even if a geometric relationship did exist for a given pair of encoders, it can be broken by a valid, equivariance-preserving transformation on one of the subjects.

1. **Initial setup:**
   - Let Person A and Person B have G-equivariant encoders, $f_A : X \to Y_A$ and $f_B : X \to Y_B$, respectively. Y_A and Y_B are neural representation spaces (Euclidean spaces $\mathbb{R}^{d_A}$ and $\mathbb{R}^{d_B}$), and the encoders induce representations $\rho_A : G \to GL(Y_A)$ and $\rho_B : G \to GL(Y_B)$.
   - Let $s_A : Q_A \to Y_A$ and $s_B : Q_B \to Y_B$ be smooth global sections for each subject's representation. Their images, $S_A = s_A(Q_A)$ and $S_B = s_B(Q_B)$, are embedded submanifolds of Y_A and Y_B, respectively. These submanifolds inherit a Riemannian metric from the Euclidean metric of their ambient spaces.

2. **Assume a relationship exists:** For the sake of argument, suppose that for a specific choice of encoders and sections, the resulting manifolds S_A and S_B happen to be isometric. This means there exists a map $\Phi : S_A \to S_B$ such that for any two points $y_1, y_2 \in S_A$, their squared distance is preserved: $||\Phi(y_1) - \Phi(y_2)||^2 = ||y_1 - y_2||^2$.
3. **Apply a plasticity transformation:**
   - According to Proposition S5, Person A can modify their encoder by composing it with a valid G-equivariant linear map $L_A : Y_A \to Y_A$ to form a new valid encoder, $\tilde{f}_A = L_A \circ f_A$.
   - This transformation maps the original section S_A to a new submanifold $\tilde{S}_A = L_A(S_A)$. The new submanifold $\tilde{S}_A$ is a valid section for the new representation space defined by the encoder $\tilde{f}_A$.
4. **The relationship is broken:**
   - Now, consider the geometry of the new section $\tilde{S}_A$. Let y_1, y_2 be two points in the original section S_A. Their images in the new section are $\tilde{y}_1 = L_A(y_1)$ and $\tilde{y}_2 = L_A(y_2)$. The squared distance between these new points is: $||\tilde{y}_1 - \tilde{y}_2||_2^2 = ||L_A(y_1) - L_A(y_2)||_2^2 = ||L_A(y_1 - y_2)||_2^2$.
   - As established in the proof of Proposition S5, if the representation ρ_A is reducible, a G-equivariant linear map L_A can be chosen such that it is not a simple scalar multiple of the identity or a rotation. Specifically, L_A can scale different irreducible subspaces of Y_A by different factors.
   - In such a case, there is no guarantee that $||L_A(y_1 - y_2)||^2 = ||y_1 - y_2||^2$. Since the internal distances within the section S_A have been altered by the map L_A, the new section $\tilde{S}_A$ is generally not isometric to the original section S_A. Consequently, it cannot be isometric to S_B either (unless by sheer coincidence, which would not constitute a "guaranteed relationship").

Since for any given pair of encoders, one can find a valid subsequent transformation for one subject that changes the intrinsic geometry of their section, no specific geometric relationship (isometry, similarity, or even diffeomorphism) between the sections of two different subjects is guaranteed by G-equivariance alone. The geometry of the section is a plastic, learnable feature of the representation. □

Proposition S7 (Intra-subject quotient diffeomorphism)**.** *Let $s_1 : Q_Y \to Y$ be a smooth global section, and let $S_1 = s_1(Q_Y)$ be its image. For any group element $g \in G$, let S_2 be the image of the section $s_2 = \rho(g) \circ s_1$. The map $\Psi_g : S_1 \to S_2$ defined by restricting the group action, $\Psi_g(y) = \rho(g)y$, is a diffeomorphism.*

Proof. To prove this, we will demonstrate that the map Ψ_g is a bijective smooth map with a smooth inverse, which are the defining characteristics of a diffeomorphism. This directly follows from Ψ_g being a restriction of a G-action, where each $\rho(g)$ is an invertible linear transformation, and such transformations are known to be diffeomorphisms.

1. **Preliminaries: smooth manifold structure of Q_Y, S_1, and S_2:**
   - The framework presupposes that Q_Y is a smooth manifold. The existence of a smooth global section $s_1 : Q_Y \to Y$ is consistent with the principal bundle being smoothly trivial.
   - For $S_1 = s_1(Q_Y)$ to be an embedded submanifold of Y, it must be the image of a smooth embedding. A smooth global section s_1 is typically defined as a smooth map such that $\pi \circ s_1 = \mathrm{Id}_{Q_Y}$. For s_1 to induce an embedded submanifold structure on its image, it must be a smooth injective immersion that maps Q_Y homeomorphically onto its image with the subspace topology. We assume s_1 satisfies these conditions, thus S_1 is a smooth manifold.
   - The map $L_g : Y \to Y$ defined by $L_g(y) = \rho(g)y$ is smooth, as $\rho : G \times Y \to Y$ is a smooth group action (a fundamental property of Lie groups and their representations). The section s_2 is the composition of two smooth maps, L_g and s_1. Therefore, $s_2 = L_g \circ s_1$ is also a smooth map. Since L_g is an invertible linear transformation (as $\rho(g) \in GL(Y)$), it is a diffeomorphism of Y onto itself. Consequently, s_2 is also a smooth embedding, making $S_2 = s_2(Q_Y)$ an embedded submanifold of Y, and thus a smooth manifold.
2. **Smoothness of Ψ_g:** The map $\Psi_g : S_1 \to Y$ is the restriction of the smooth map $L_g : Y \to Y$ to the smooth embedded submanifold S_1. The restriction of a smooth map to a smooth submanifold is smooth (Theorem 5.53(a) in [3]). Therefore, $\Psi_g : S_1 \to Y$ is smooth. Since its image is precisely S_2, Ψ_g can be considered as a smooth map from S_1 to S_2.
3. **Bijectivity of Ψ_g:**
   - **Injectivity:** Suppose $\Psi_g(y_1) = \Psi_g(y_2)$ for $y_1, y_2 \in S_1$. This means $\rho(g)y_1 = \rho(g)y_2$. As ρ is a linear representation of the Lie group G, each $\rho(g)$ is an invertible linear transformation (an element of $GL(Y)$). Thus, $\rho(g)$ has an inverse, which is $\rho(g^{-1})$. Applying $\rho(g^{-1})$ to both sides of the equation yields $\rho(g^{-1})\rho(g)y_1 = \rho(g^{-1})\rho(g)y_2$. By the group homomorphism property, $\rho(g^{-1})\rho(g) = \rho(g^{-1}g) = \rho(e)$, where e is the identity element of G. The identity element of a group representation maps to the identity linear transformation, i.e., $\rho(e) = \mathrm{Id}_Y$. Therefore, $y_1 = y_2$. This proves Ψ_g is injective.
   - **Surjectivity**: Let $z \in S_2$ be an arbitrary point. By the definition of S_2, there exists a point $q \in Q_Y$ such that $z = s_2(q)$. Since $s_2 = \rho(g) \circ s_1$, we can write $z = \rho(g)(s_1(q))$. Let $y_1 = s_1(q)$. By definition of S_1, $y_1 \in S_1$. Substituting this into the expression for Ψ_g, we find $\Psi_g(y_1) = \rho(g)y_1 = \rho(g)(s_1(q)) = z$. This proves Ψ_g is surjective.
   - Since Ψ_g is both injective and surjective, it is a bijection.
4. **Smoothness of the Inverse Map Ψ_g^{-1}:**
   - The inverse map $\Psi_g^{-1} : S_2 \to S_1$ is given by $\Psi_g^{-1}(z) = \rho(g^{-1})z$.
   - Analogous to the argument for Ψ_g itself, this inverse map is also a restriction of a smooth map ($L_{g^{-1}}(z) = \rho(g^{-1})z$) to a smooth embedded submanifold (S_2). Therefore, Ψ_g^{-1} is also smooth.

Since $\Psi_g : S_1 \to S_2$ is a smooth bijective map with a smooth inverse, it satisfies the definition of a diffeomorphism. □

Compositionality and hierarchical structures of qualia

While our framework mainly focused on the geometric structure induced by a single symmetry group, G, conscious experience is often compositional, involving multiple interacting symmetries. How these different symmetries combine to create unified, structured experiences is a key question. Here, we extend our framework to model this compositionality using two fundamental algebraic structures: the direct product, which describes independent, disentangled qualia attributes, and the semidirect product, which describes hierarchically compositional, entangled qualia attributes.

For perceptually independent features, the total symmetry can be modeled as a direct product of groups ($G_{\text{total}} = G_1 \times G_2$). This structure corresponds to a direct product of principal bundles, a concept central to disentangled representation learning in AI [5, 6, 7, 8, 9]. In such a system, where the group $G_1 \times G_2$ acts component-wise on the product space $Y_1 \times Y_2$, the quotient space of signatures naturally factorizes into a product of the individual quotient spaces (Figure 7A in the main text):

$$(Y_1 \times Y_2)/(G_1 \times G_2) \cong (Y_1/G_1) \times (Y_2/G_2) =: Q_1 \times Q_2 \quad \text{(S14)}$$

This factorization provides a formal model for how a neural system can, in principle, separate independent qualia attributes. At the highest level, the total experiential space Y might decompose into independent modalities such as vision and audition ($Y \cong Y_v \times Y_a$). Even within a single modality, a network could learn perfectly disentangled representations for an object's pose and color ($G_v \cong G_{\text{pose}} \times G_{\text{color}}$), where the qualia attributes of pose and color could be experienced independently and commutatively (Figure 7B in the main text). However, achieving such ideal factorization in practice remains a significant challenge [10, 11].

While the direct product is a useful idealization, a unified percept often requires tighter, hierarchical integration of features. This feature entanglement is modeled by the semidirect product, $G_{\text{total}} = G_1 \rtimes_\phi G_2$, defined by the group operation $(g_1, g_2) \cdot (g_1', g_2') = (g_1 \cdot \phi_{g_2}(g_1'), g_2 \cdot g_2')$, where $\phi : G_2 \to \mathrm{Aut}(G_1)$ is a homomorphism specifying how G_2 elements modify those of G_1. This structure is fundamentally non-commutative, reflecting hierarchical dependence: G_2 actions alter G_1's effective transformations, but not vice versa. Geometrically, it induces a composite bundle, a hierarchy of bundles, with a two-stage projection [12]:

$$Y \to Q_1 \to Q_{12}. \quad \text{(S15)}$$

The total space Y first forms a bundle under the action of the group G_1, which partitions Y into orbits and yields the quotient space Q_1. Then, this quotient space Q_1 itself serves as a new space that is acted upon by the second group, G_2. This second action organizes Q_1 into another bundle structure, yielding a final quotient space, Q_{12}. A classic example is the combination of object translation ($G_1 = \mathbb{R}^2$) and rotation ($G_2 = SO(2)$) (Figure 7C in the main text), forming the special Euclidean group $SE(2)$. Perceptually, rotating an object changes its effective position non-commutatively; that is, translating then rotating differs from rotating then translating. This mirrors how visual stability (e.g., object constancy under motion) feels "entangled," with position attributes depending on orientation context.

In summary, these two algebraic structures provide a richer language for modeling different modes of qualia composition. The direct product models the independence of qualia attributes through a commutative structure, corresponding geometrically to a simple, factorizable bundle ideal for disentangled experiences. In contrast, the semidirect product models the dependence and entanglement of qualia attributes, leading to a hierarchical, composite bundle geometry that explains how different attributes can be woven together into a single, coherent, and unified conscious experience.

References

1. Brian C Hall. *Lie groups, lie algebras, and representations: An elementary introduction*. Graduate texts in mathematics. Springer International Publishing, Cham, Switzerland, 2 edition, May 2015.
2. Norman Steenrod. *The topology of fibre bundles. (PMS-14)*. Princeton Mathematical Series. Princeton University Press, Princeton, NJ, January 1999.
3. John M Lee. *Introduction to Smooth Manifolds*. Graduate texts in mathematics. Springer New York, New York, NY, August 2012.
4. Jean-Pierre Serre. *Linear representations of finite groups*. Graduate texts in mathematics. Springer, New York, NY, September 1977.
5. Irina Higgins, David Amos, David Pfau, Sebastien Racaniere, Loic Matthey, Danilo Rezende, and Alexander Lerchner. Towards a definition of disentangled representations. *arXiv [cs.LG]*, December 2018.
6. Robin Quessard, T Barrett, and William R Clements. Learning disentangled representations and group structure of dynamical environments. In H Larochelle, M Ranzato, R Hadsell, M F Balcan, and H Lin, editors, *Advances in Neural Information Processing Systems*, volume 33, pages 19727–19737. Curran Associates, Inc., December 2020.
7. Hugo Caselles-Dupré, Michael Garcia Ortiz, and David Filliat. Symmetry-based disentangled representation learning requires interaction with environments. In *Advances in Neural Information Processing Systems*, volume 32, pages 4606–4615. Curran Associates, Inc., 2019.
8. Xiao Liu, Pedro Sanchez, Spyridon Thermos, Alison Q O'Neil, and Sotirios A Tsaftaris. Learning disentangled representations in the imaging domain. *Med. Image Anal.*, 80(102516):102516, August 2022.
9. Xin Wang, Hong Chen, Si'ao Tang, Zihao Wu, and Wenwu Zhu. Disentangled representation learning. *IEEE Trans. Pattern Anal. Mach. Intell.*, 46(12):9677–9696, December 2024.
10. Irina Higgins, Sébastien Racanière, and Danilo Rezende. Symmetry-based representations for artificial and biological general intelligence. *Front. Comput. Neurosci.*, 16:836498, April 2022.
11. Francesco Locatello, Stefan Bauer, Mario Lucic, S Gelly, B Scholkopf, and Olivier Bachem. Challenging common assumptions in the unsupervised learning of disentangled representations. In Kamalika Chaudhuri and Ruslan Salakhutdinov, editors, *Proceedings of the 36th International Conference on Machine Learning*, volume 97 of *Proceedings of Machine Learning Research*, pages 4114–4124. PMLR, June 2019.
12. Gennadi Sardanashvily. *Advanced differential geometry for theoreticians: fiber bundles, jet manifolds and Lagrangian theory*. LAP Lambert Academic Publishing AG, Saarbrucken, Germany, May 2013.
